# Nanoscale isoindigo-carriers: self-assembly and tunable properties

**DOI:** 10.3762/bjnano.8.34

**Published:** 2017-02-01

**Authors:** Tatiana N Pashirova, Andrei V Bogdanov, Lenar I Musin, Julia K Voronina, Irek R Nizameev, Marsil K Kadirov, Vladimir F Mironov, Lucia Ya Zakharova, Shamil K Latypov, Oleg G Sinyashin

**Affiliations:** 1 A.E. Arbuzov Institute of Organic and Physical Chemistry, Kazan Scientific Center, Russian Academy of Sciences, Arbuzov str. 8, Kazan, 420088, Russian Federation

**Keywords:** drug delivery systems, dyes, isoindigo, nanoparticles, self-assembly, surfactants

## Abstract

Over the last decade isoindigo derivatives have attracted much attention due to their high potential in pharmacy and in the chemistry of materials. In addition, isoindigo derivatives can be modified to form supramolecular structures with tunable morphologies for the use in drug delivery. Amphiphilic long-chain dialkylated isoindigos have the ability to form stable solid nanoparticles via a simple nanoprecipitation technique. Their self-assembly was investigated using tensiometry, dynamic light scattering, spectrophotometry, and fluorometry. The critical association concentrations and aggregate sizes were measured. The hydrophilic–lipophilic balance of alkylated isoindigo derivatives strongly influences aggregate morphology. In the case of short-chain dialkylated isoindigo derivatives, supramolecular polymers of 200 to 700 nm were formed. For long-chain dialkylated isoindigo derivatives, micellar aggregates of 100 to 200 nm were observed. Using micellar surfactant water-soluble forms of monosubstituted 1-hexadecylisoindigo as well as 1,1′-dimethylisoindigo were prepared for the first time. The formation of mixed micellar structures of different types in micellar anionic surfactant solutions (sodium dodecyl sulfate) was determined. These findings are of practical importance and are of potential interest for the design of drug delivery systems and new nanomaterials.

## Introduction

Studies of amphiphilic isoindigo derivatives have revealed perspectives and high potential in several fields: (i) Supramolecular assemblies of amphiphilic conjugated π-systems can potentially be useful structures with improved tunable optical and electrical properties [1–5]. This makes possible to develop new technologies for biodiagnostics, biomedical applications, including photothermal therapy [6–8], study of biomembranes [9] and dynamically probing cells [10–11]. (ii) These heterocyclic compounds found applications in various pharmaceutical and synthetic protocols [12–21]. It is known that isatin and isoindigo derivatives possess antibacterial [12,22], antifungal [23–24], and antiviral activity [25–26]; they are promising platforms for the design of anti-HIV agents [27] and they are also used in the treatment of leukaemia [28–32]. (iii) Another advantage of isatin and isoindigo derivatives is that they display binding properties of biomacromolecules (DNA, proteins and enzymes). It is noteworthy that isatin derivatives interact with DNA via an intercalating mechanism [12–13,33]. This may be of potential interest in biomedicine for the delivery of drugs or genetic material into cells [34–35].

The development of effective therapeutic drugs based on isoindigo derivatives focuses on improving their bioavailability because of their weak solubility in water. To this end an isoindigo scaffold was functionalized with carbohydrate substituents [36–37]. Over the last three decades, extensive research in nanomedicine led to several strategies to improve drug biocompatibility and drug delivery efficacy (e.g., synthesis of polymer–drug conjugates, such as polyethylene glycol, hyaluronic acid, and heparin, also amphiphilic prodrugs, and supramolecular hydrogels) [38–40]. One of the main strategies deals with the creation of self-assembled supramolecular structures with tunable morphologies (e.g., nanospheres, rods, nanofibers or nanotubes) adapted to the administration route. Drug nanostructures thus obtained are single-component systems unlike traditional colloidal drug carriers. The design of building blocks allows for the control of physical and chemical properties of self-assembled systems. Self-assembly [41] and nanoprecipitation [42] are the most common approaches to create nanostructures [43]. Unlike traditional low-molecular surfactants, e.g., π-functional amphiphiles, the self-assembly of amphiphilic drugs bearing a chromophoric moiety can produce more robust self-assemblies [44–45]. Different approaches can be used to produce various supramolecular systems [46–48]. These strong interactions are critical for the biological function of drugs [49]. In our previous work we described the ability of an amphiphilic isoindigo derivative bearing octadecyl substituents to form self-assembled structures of micellar type in water/DMF solution [50]. In the present work, we focused on the fabrication of nanoscale isoindigo carriers using two approaches: self-assembly and nanoprecipitation. The study aimed to demonstrate the possibility to modulate drug delivery of amphiphilic isoindigo derivatives by tuning hydrophilic–lipophilic balance, π–π stacking interaction and hydrogen bonding.

Another strategy is the use of soft matter (micelles, emulsions, dendrimers, nanospheres, solid lipid nanoparticles or liposomes) as the delivery vehicle. These studies have been encouraged by the possibility to prevent side effects, to increase drug bioavailability, to decrease toxicity as well as to minimize drug degradation and to provide a controllable drug release [51–53]. The modification of nanostructures with conjugated π–π fragments leads to the absorption of anticancer drugs via π–π stacking interaction and increases the drug-loading capacity of nanoscale soft materials [54]. The latter feature is essential for designing novel antitumor drugs.

**Figure 1:**
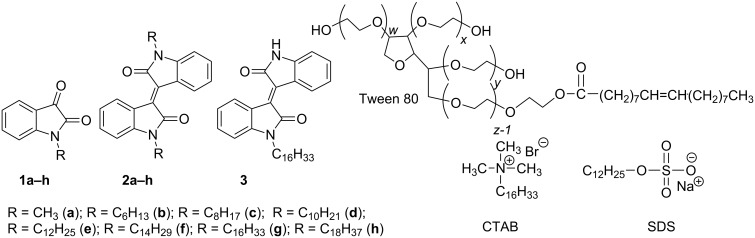
Structures of isatin (**1a–h**), isoindigo derivatives **2a–h**, **3** and anionic (sodium dodecyl sulfate, SDS), cationic (cetyltrimethylammonium bromide, CTAB) and nonionic (Tween 80) surfactants.

The second stage of the work was devoted to solubilize amphiphilic isoindigo derivatives using surfactants that are widely used in pharmaceutical industry. The aim of the present work was to investigate the ability of surfactants to bind isoindigo derivatives. For this purpose were used three different surfactants and amphiphilic isoindigo compounds with different lengths of the alkyl substituent at the endocyclic nitrogen atom (Figure 1).

## Results and Discussion

### Nanoscale isoindigo carriers: self-assembly and solid nanoparticles

For the preparation of solid isoindigo nanoparticles (SIPs), the method of nanoprecipitation was used. Precipitation is a commonly used technique in pharmaceutical technology [42,55–56]. This method is documented for protein encapsulation [57], hydrophobic drugs [58], and also for targeting cancer cells [59]. The factors controlling the formation of nanoparticles have been determined [42,57]. Nanoprecipitation is ideal, when the compound must dissolve in one component (the solvent), but must not in the second one (the non-solvent). This one-step manufacturing process commonly available to prepare nanoparticles from polymers that was already reviewed [57,60–61]. In recent years, amphiphilic cyclodextrins [62], calix[*n*]arenes and calix[*n*]resorcinarenes [63] were used in the formation of nanoparticles via a simple nanoprecipitation technique. The influence of the hydrophobicity of the drug (paclitaxel) on the therapeutic efficacy was also shown [64].

**Figure 2:**
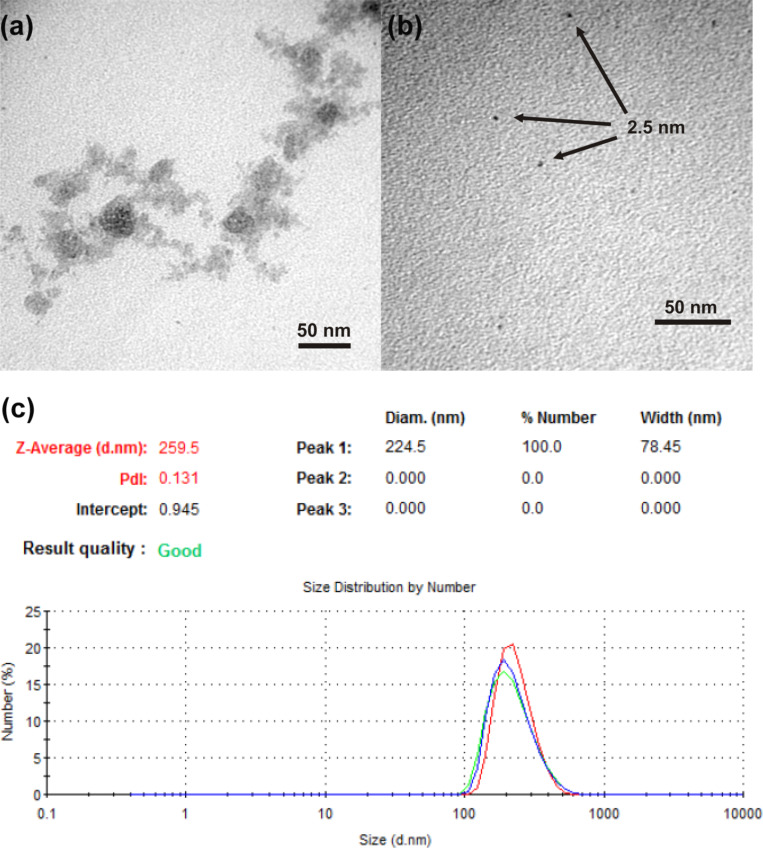
Transmission electron micrographs (TEM) (a,b); histogram of the particle size distribution (c) of **2h** solid isoindigo nanoparticles (SIPs).

As seen in Figure 2a, the size of SIPs of **2h** is ca. 300 nm, which is comparable with that determined by DLS (Figure 2c). The morphology of aggregates resembles branched elongated structures consisting of small particles of 2–3 nm (Figure 2b). The sizes of SIPs of **2b**, **2d**, **2e**, **2f**, **2h** are given in Table S1 (Supporting Information File 1). The long-chain homologues **2g** and **2h** have smaller sizes of 150 to 300 nm and a low polydispersity index (0.15 ± 0.02). They are rather stable, i.e., the size and polydispersity index change only little over time at room temperature (for more than 80 days). The zeta potentials of SIPs from **2d**, **2e**, **2f**, **2h** are about −34, −28, −30, −39 mV, respectively (Table S1, Figure S1, Supporting Information File 1). To characterize the colloidal stability of the compounds under study in the presence of electrolytes, the stability of these fabricated SIPs was evaluated under in vitro conditions. After 3 h dialysis in phosphate buffer (pH 7.4) at 37 °C the size of **2d** SIPs was shown to increase, with the polydispersity index reaching values greater than 0.4 (Figure S2, Supporting Information File 1). Homologues **2b** and **2e** form large aggregates of 500 to 700 nm in size. We failed to prepare nanoparticles for high homologues **1g** and **3** through the nanoprecipitation technique. It is known that size and morphology of aggregates in aqueous solutions are markedly determined by hydrophilic-lipophilic balance. This is probably due to the packing parameter [65] of compounds **2**. Unlike derivatives **1g** and **3**, a series of compounds **2** tends to form layered self-assembled structures, as described further.

### Size and morphology of isoindigo supramolecular structures

**Figure 3:**
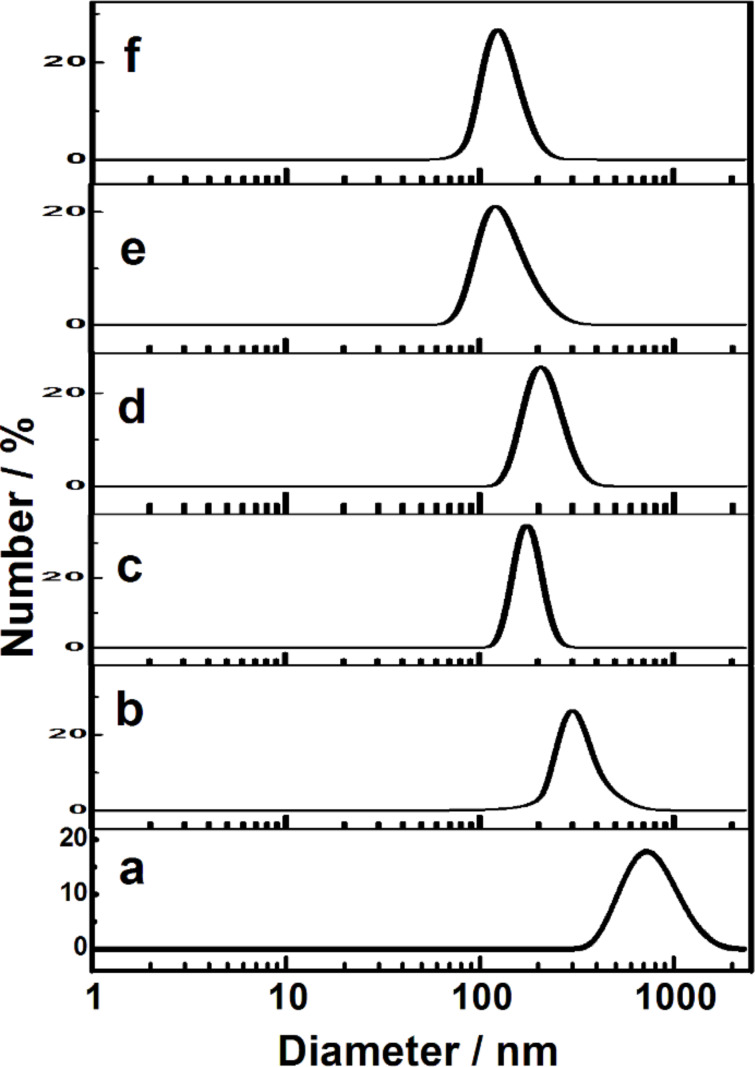
Analysis of the size distribution of **2c** (a), **2d** (b), **2e** (c), **2f** (d), **2g** (e), **2h** (f) particles in water/DMF (50% v/v) solutions using the number parameter, *c* = 1 mM, 25 °C.

The DLS study of isoindigo derivatives demonstrated the ability to self-assemble in solution. In Figure 3 and Table S2 (Supporting Information File 1), aggregate sizes and polydispersity index at various isoindigo concentrations are given.

As seen, aggregate size depends on the length of hydrocarbon fragments of isoindigo with the particle sizes of compounds **2с**–**f** varying within the range of 200 to 700 nm. The long-chain homologues **2g** and **2h** form particles of 100 to 200 nm. The sizes tend to increase with the increase in concentration of isoindigo derivatives (Figure S3 and Figure S4, Supporting Information File 1). The polydispersity index is rather low (0.2) and changes only little with increasing concentration (Table S2, Supporting Information File 1). The zeta potential of **2h** particles is about −30 ± 1 mV (Figure S5, Supporting Information File 1). The increase in temperature up to 50 °С is shown to result in the breaking of the particles of the short-chain derivative **2c**. This behaviour may be caused by the effect of temperature on hydrogen bonds and/or π-stacking interaction of aggregated **2с**. In the case of the long-chain homologue **2h**, the increase in temperature exerted little effect on aggregation (Figure S6, Supporting Information File 1). The size of the **2h** aggregates increased when using phosphate buffer instead of water during preparation of the colloidal aggregates (Figure S7, Supporting Information File 1).

To predict the morphology of aggregates, the packing parameter *P* [65] was calculated for all compounds by using Equation 1:

[1]
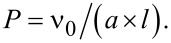


The dimensionless quantity *P* is the ratio between the volume of the hydrophobic fragment, v_0_ and the polar head surface area, *a*, multiplied by the chain length of the hydrophobic fragment, *l*. For the cone-like amphiphilic molecules, the value of *P* of which is below 1/3, the formation of spherical aggregates may be expected. This is observed in the case of typical surfactants bearing relative large polar fragment and single alkyl chain. If 1/2 < *P* ≤ 1, the formation of bilayers is predicted, including closed structures, i.e., vesicles.

The polar head surface area was calculated as the sum of the area of hexagons and pentagons based on the XRD data for isoindigo derivatives available in the Cambridge Structural Database. As seen in Figure S8 (Supporting Information File 1), the surface of the oxindole fragment of isoindigo derivatives, for which the crystal structure is currently known is in the range of 0.84–0.87 nm^2^. The dimensions of the oxindole fragment for compounds **1g**, **2c**, and **2d** are 0.86, 0.86 and 0.85 nm^2^, respectively, (Table S3; Supporting Information File 1). All of these values are very close. The values of v_0_ and *l* for alkyl groups can be calculated using the relationships in Equation 2 and Equation 3 [66]:

[2]
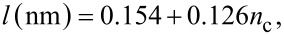


[3]
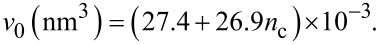


Data in Table S3 (Supporting Information File 1) show that packing parameters (*P*) are 0.25 and 0.12 for compounds **1g** and **3**, respectively, which makes it plausible to assume the occurrence of spherical normal micelles. For derivatives **2a–h** *P* values are close to 0.5, which indicates the formation of aggregates with lower surface curvatures, e.g., layered structures.

### CAC and other aggregation parameters

The structure of the long-chain homologues of isatin (**1g**) and isoindigo (**2e**, **2g**, **2h** and **3**) contains hydrocarbon fragments and is similar to that of typical surfactants. The self-assembly of amphiphilic molecules bearing a chromophoric moiety is somewhat different from that of classical surfactants. For example, π-functional amphiphiles can yield a more robust self-assembly [44–45]. Nevertheless, we tried to determine the critical association concentration (CAC) and other aggregation parameters. Initially we studied the ability of compounds to localize at the air–solvent interface. A water/DMF (50% v/v) mixture was used as solvent. Unfortunately, we failed to prepare stable solutions of **1g** and **3**. Tensiometry experiments allowed us to determine that the amphiphilic isoindigo derivatives **2e**, **2g**, **2h** do not decrease the surface tension of the solvent (52 mN/m). They are surface-inactive compounds (Figure S9, Supporting Information File 1).

**Figure 4:**
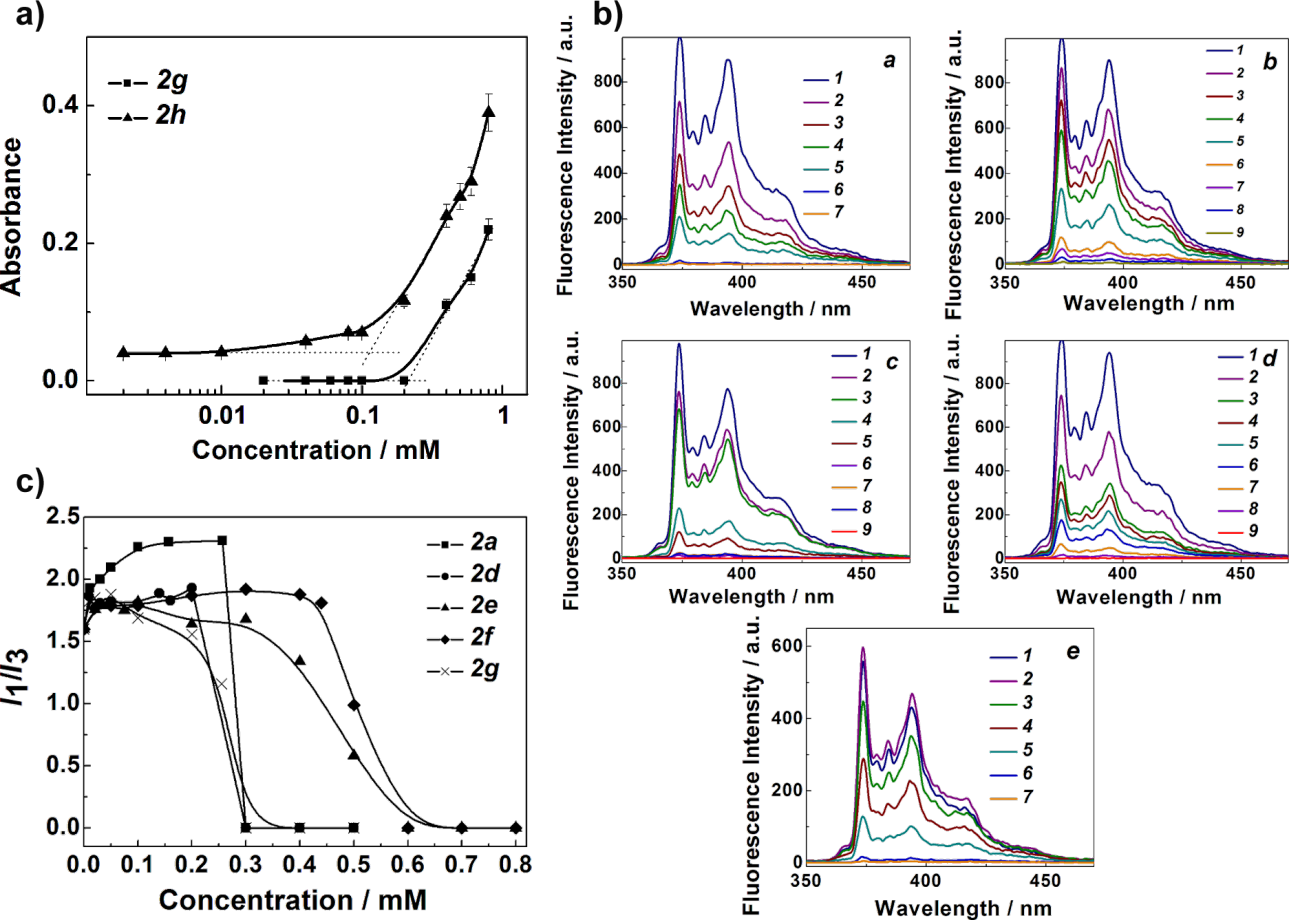
(a) Absorbance of the dye (Sudan I) at 495 nm as a function of the concentration of **2g** and **2h** in water/DMF (50% v/v) solution, 25 °C; optical length 0.1 cm. (b) Fluorescence of pyrene (*c*_pyrene_ = 1 × 10^−6^ M) in **2a** (a), **2d** (b), **2e** (c), **2f** (d), and **2g** (e) in water/DMF (50% v/v) solution, 25 °C, *c***_2a_** (mM): 0 (1)–0.257 (7); *c***_2d_** (mM): 0 (1)–0.2 (9); *c***_2e_** (mM): 0 (1)–0.5 (9); *c***_2f_** (mM): 0 (1)–0.5 (9); *c***_2g_** (mM): 0.0097 (1)–0.255 (7). (c) Dependence of the intensity ratio (*I*_1_/*I*_3_) of the first and third peaks of pyrene on the **2a**, **2d–g** concentrations, *c*_pyrene_ = 1 × 10^−6^ M, 25 °C.

The study of the entrapment of hydrophobic probes is a generally accepted assay for the potential of soft materials to be used as nanocontainers for hydropohobic solutes including drugs. Furthermore, the solubilization of organic hydrophobic dyes makes it possible to detect the formation of micelles in solution [67]. In Figure 4a, the dependence of the absorbance of hydrophobic dye (Sudan I) on the concentration of isoindigo derivatives **2g** and **2h** is shown. The samples based on compounds **2a–f** were not used in this experiment due to their low stability in the presence of Sudan I. In these cases, gradual agglomeration and precipitation were observed in the systems.

As seen in Figure 4a an increase in absorbance occurs at concentrations above 0.2 mМ (for **2g**) and 0.1 mМ (for **2h**). Thus, experimental data prove the formation of self-assembled structures of **2g** and **2h** with a hydrophobic field that can dissolve nonpolar probes. The critical association concentrations for **2g** and **2h** are 0.2 and 0.1 mМ, respectively. Calculated values of the solubilization capacity of **2g** and **2h** micellar aggregates are given in Table S4 (Supporting Information File 1). As can be seen, the solubilization capacity value grows with the increasing length of the alkyl chain. According to Table S4 (Supporting Information File 1), the solubilization ability of **2g** and **2h** is, respectively, two times and 20 times higher than that of the classical surfactants, CTAB and SDS.

Fluorescence studies showed that significant changes occur in the pyrene spectrum of the isoindigo derivatives in solution for all the compounds under study, regardless the length of the alkyl chain (Figure 4b). The change in pyrene fluorescence intensity is likely due to the decrease in the polarity of the pyrene microenvironment. Based on these spectra, the CAC of amphiphilic compounds was calculated from the *I*_1_/*I*_3_ values in the pyrene fluorescence spectrum as a function of the concentration of amphiphilic compounds [68–69]. Most probably, pyrene is incorporated into the hydrophobic region of the colloidal aggregates. Usually, the CMC values of surfactants determined by different methods are very similar [70]. But an additional binding of the fluorescent probe (pyrene) to the heterocyclic core of the isoindigo compounds cannot be excluded. Therefore, we recognize that some perturbation of micelles can occur. On the other hand, the latter can be neglected because of the very low pyrene concentration used. In our case, according to data given in Figure 4c, the CAC of compound **2g** is 0.3 mM, which is comparable with that determined spectrophotometrically (Figure 4a). Compounds **2e** and **2f** aggregate at higher concentrations (CAC ≈ 0.6 mМ). Importantly, this rather low aggregation number may result from the quenching effect of isoindigo derivatives [50]. Figure 4b and Figure S10 (Supporting Information File 1) (Stern–Volmer dependences) show that the most effective quenching occurs for compounds **2a**, **2d** and **2g**. The short-chain isoindigo derivatives **2a–d** presumably form supramolecular polymers or layered particles, which can effectively quench the pyrene fluorescence. These structures can be formed through intermolecular π-stacking interactions and/or the formation of hydrogen bonds. The literature data provide information that some isoindigo derivatives and other chromophores tend to form J-aggregates in solid state [71].

**Scheme 1:**
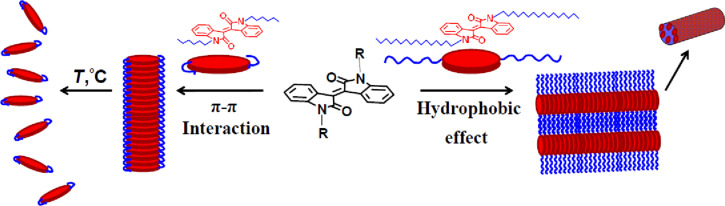
Supramolecular assemblies of isoindigo derivatives **2a–h**.

One can assume that short-chain isoindigo derivatives form layered supramolecular aggregates largely through π–π stacking interactions, while the aggregation of the long-chain homologues is mainly guided by hydrophobic effects (Scheme 1).

### Micellar solutions of isoindigo derivatives and their spectroscopic properties

One way to improve the bioavailability of isoindigo derivatives is the formation of mixed supramolecular assemblies with various types of surfactants. Micellar solubilization is one approach for the improvement of solubility of hydrophobic drugs [72]. It is known that the incorporation of poorly water-soluble drugs in the nonpolar core of micelles may increase solubility, stability, and bioavailability [73–75]. Importantly, amphiphilic molecules and drugs can form mixed assemblies that would result in changing in the micellization process [66]. In addition, the amphiphilic nature of surfactant micelles can serve as a tentative model of biomembranes [74,76]. Thus, the study of interactions with various surfactants may provide a deeper insight into the transport and binding of isatin and isoindigo derivatives at the molecular level. We have studied the effect of the structure of isatin and isoindigo derivatives on the solubilizing properties of commercially available classical anionic (SDS), cationic (CTAB) and nonionic (Tween 80) surfactants.

**Figure 5:**
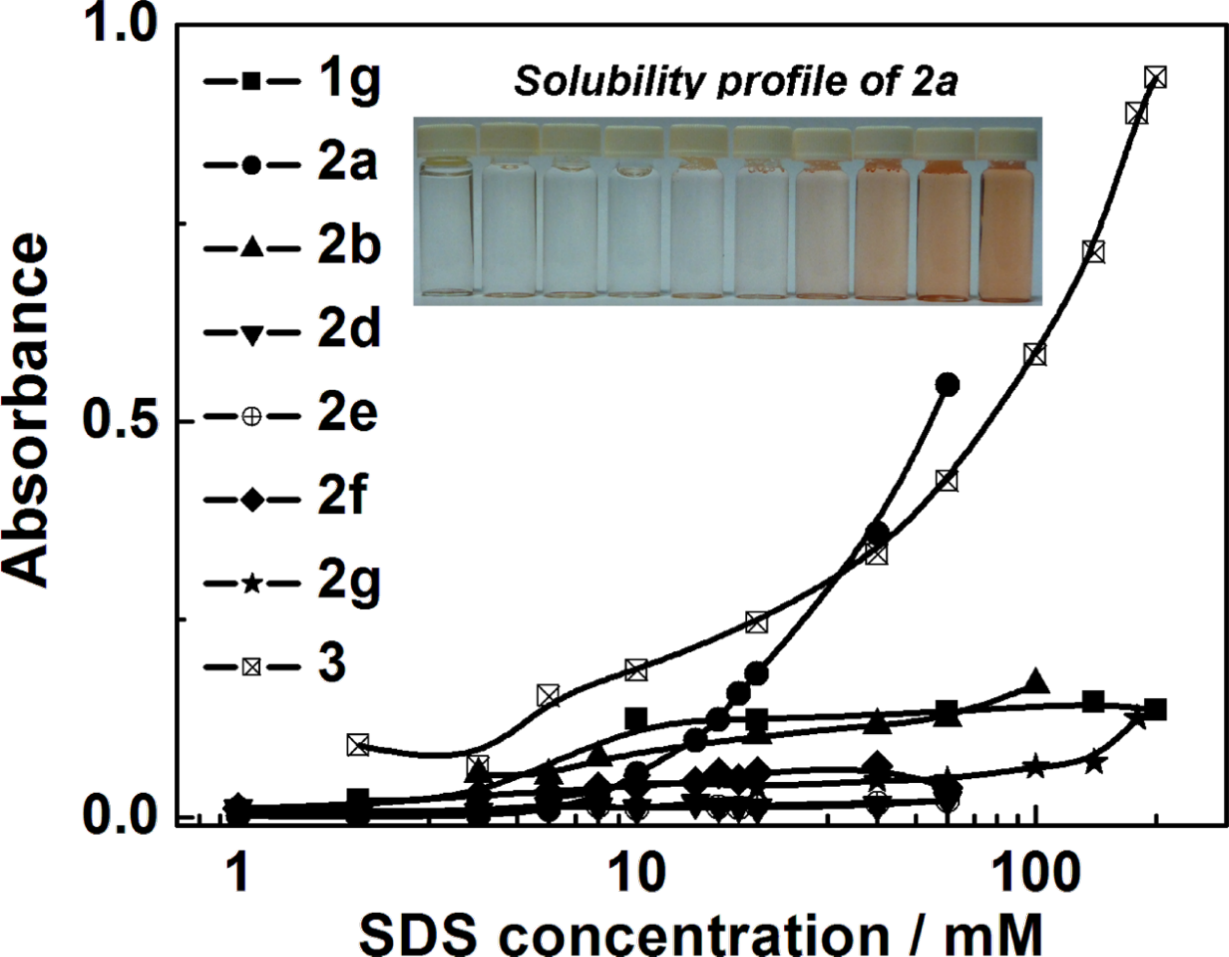
Absorbance of **2a**, **2b**, **2d–g** at λ_max_ ≈ 395 nm and **1g**, **3** at λ_max_ = 300 nm in aqueous micellar SDS solution; 25 °C, optical path 1 cm and solubility profile solution of SDS in the presence of **2a** on concentration of SDS, *c*_SDS_= 2–80 mM, 25 °C.

Because isatin and isoindigo derivatives are new dyes, their extinction coefficients were determined for the first time. UV–vis absorption spectra of **2a–h** and **3** in chloroform are presented in Figures S11–S18 (Supporting Information File 1). The absorption spectra of **1g** in different solvents (CHCl_3_, EtOH and DMF, Figures S19–S21, Supporting Information File 1) were also recorded. The maximum absorption wavelength of **1g** (λ_max_ = 300 nm) does not vary significantly with the type of solvent. The extinction coefficients of the studied compounds are presented in Table S5 (Supporting Information File 1). In Figure 5, the dependence of the absorbance of isatin and isoindigo derivatives in aqueous SDS solution on the SDS concentration is given.

The data show that the isoindigo derivatives **2a** and **3** are most effectively solubilized in SDS micelles. For isoindigo derivatives with two alkyl tails, the highest solubilization occurs for compound **2a**, i.e. for isoindigo with two methyl groups. It is important to note that unlike the other tested compounds, **2a** has the highest extinction coefficient of ε = 13447 M^−1^·cm^−1^ (λ_max_ = 395 nm, Table S5, Supporting Information File 1). Although compound **2e** has a similar value of ε = 13156 M^−1^·cm^−1^ (λ_max_ = 395 nm), it is less effectively solubilized in SDS micelles. In the case of compound **1g**, solubilizing capacity cannot be estimated because its extinction coefficient is only about one sixth of that of isoindigo **2a**. The solubilization of compound **3** is observed in micellar solutions of CTAB and Tween 80 (Figure S23 and Figure S24, Supporting Information File 1).

**Figure 6:**
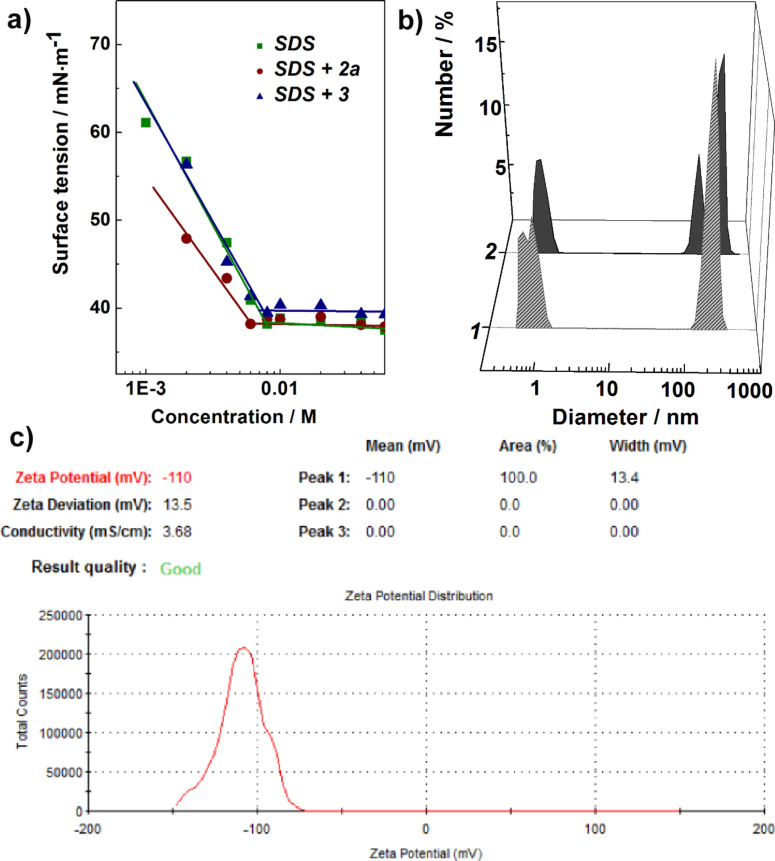
(a) Surface tension isotherms of SDS in the absence and presence of **2a** and **3** in water, (b) Analysis of the size distribution and (c) zeta potential of **2a** particles in water–SDS solutions, *c*_SDS_ (mM): 60 (1), 80 (2), 25 °C.

It is noteworthy that apart from the solubilization mechanism by typical interaction between surfactants and dyes, mixed aggregates can be formed. The surface tension of SDS solutions admixed with **2а** and **3** are given in Figure 6. The formation of mixed structures of SDS and isoindigo derivative **2a** is observed in a lower concentration range compared to single SDS micelles. This is supported by the left-shift in the surface tension isotherms of SDS with **2a** additives (Figure 6a) and the decrease of the slope, indicating a lower surface activity. This is presumably related to the formation of structures with various aggregation numbers. Measurement of the size of **2а** particles in SDS solution showed the presence of particles of about 1 and about 200 nm in diameter (Figure 6b). The former most likely correspond to SDS micelles, while the latter represent mixed structures of SDS and the isoindigo derivative. In the SDS–**2a** system an increase in zeta potential is observed (Figure 6c).

**Scheme 2:**
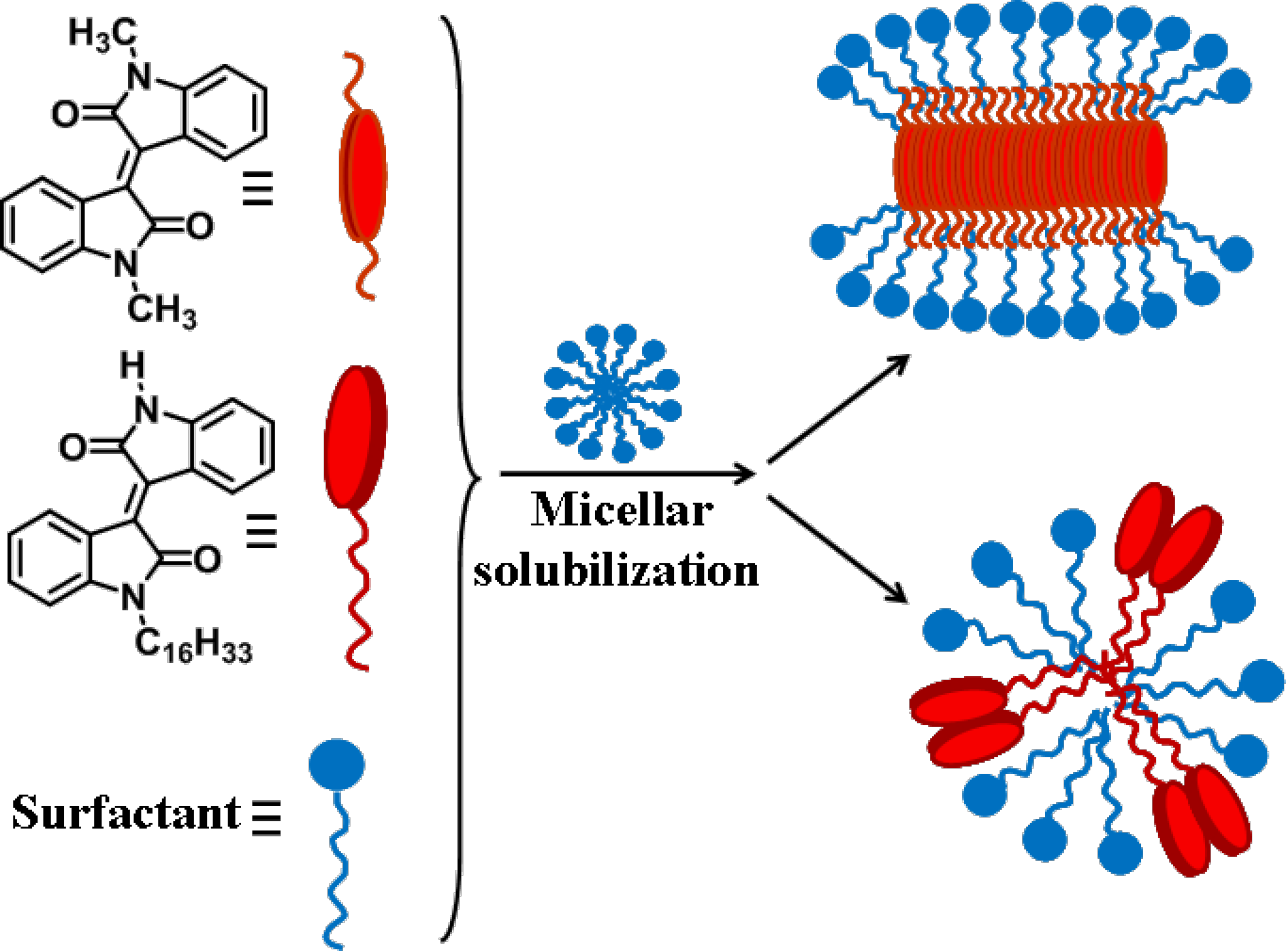
Solubilization of **2a** and **3** in SDS.

According to our results and literature data [77–78], the zeta potential of SDS micelles is about −70 mV, while the mixed aggregates of SDS and **2a** have a zeta potential of about −110 mV. In the case of compound **3**, there is a decrease in the zeta potential of SDS micelles by ca. 20 mV. Most probably, **2a** molecules are incorporated and dissolved in the nonpolar core of the SDS micelles. This may lead to a change in the packing parameter of SDS molecules, an increase in the aggregation numbers of surfactant micelles and/or the formation of non-spherical structures, and, consequently, in an increase in the zeta potential of the system. For the amphiphilic derivative **3**, the probability of the formation of micelle-like aggregates of SDS and **3** increases. This may result in a loosening of the SDS micelles, a decrease in the aggregation numbers and, hence, in a decrease in the zeta potential of the system. A schematic representation of mixed structures of SDS and isoindigo derivatives is shown in Scheme 2.

## Conclusion

Solid nanoparticles and self-assembled structures of dialkylated isoindigo derivatives were obtained. Size, morphology, and stability of supramolecular associates and solid nanoparticles of dialkylated isoindigo derivatives depend on the length of the alkyl-chain moiety. Stable solid nanoparticles were obtained for dialkylated isoindigo derivatives with C_16_H_33_, and C_18_H_37_, their size ranged between 150 and 300 nm. The size of self-assembled structures of dialkylated isoindigo derivatives was equal to 200–700 nm in the case of R = C_8_H_17_, C_10_H_21_, C_12_H_25_, C_14_H_29_; and 100–200 nm in the case of R = C_16_H_33_ and C_18_H_37_. The polydispersity index of the particles is below 0.2. The critical association concentration for dialkylated isoindigo derivatives with R = C_12_H_25_, C_14_H_29_, C_16_H_33_, and C_18_H_37_ decreases with the increase in hydrocarbon chain length and corresponds to 0.6, 0.2, and 0.1 mМ, respectively. Effective pyrene quenching is observed for all dialkylated isoindigo derivatives. This is mostly related to the formation of supramolecular polymers or layered aggregates driven by π–π interaction in the case of short-chain isoindigo derivatives and micelle-like aggregates in the case of long-chain isoindigo derivatives. The alkylated isatin and isoindigo derivatives were employed for the first time as probe dyes for various types of surfactants (cationic, anionic, and nonionic). It was spectrophotometrically determined that at surfactant (SDS, CTAB, Tween 80) concentrations above the CMC, an increase in absorption occurs for the compounds **2a** and **3**. Likely, the dissolution of short-chain dialkylated isoindigo derivative **2a** and monoalkylated isoindigo derivative **3** in micellar surfactant solutions is due to the formation of different types of mixed surfactant–isoindigo derivative assemblies. The data obtained are expected to contribute to the development of nanodevices with interesting optical properties and pharmacological applications as drug delivery systems.

## Experimental

### Materials

1-Phenylazo-2-naphthol (Sudan I, Acros Organics, New Jersey, USA), pyrene for fluorescence (Sigma, Switzerland, 99%), cetyltrimethylammonium bromide (CTAB) (Acros Organics, New Jersey, USA, 99%), sodium dodecyl sulfate (SDS) (Acros Organics, New Jersey, USA, 99%), Tween 80 (Acros Organics, New Jersey, USA, BioXtra) were used. Unless otherwise mentioned, other chemicals and solvents were of analytical grade from commercial sources. Dimethylformamide (DMF) and tetrahydrofuran (THF) were purified according to conventional procedures [79]. Compounds **1a–h**, **2a–h** were obtained as reported earlier [50].

#### Synthesis of 1-hexadecyl-(3,3′-biindolinylidene)-2,2′-dione **3**

A mixture of 1-hexadecylisatin **1g** (3.0 g, 8.1 mmol) and indolin-2-one (1.08 g, 8.1 mmol) in acetic acid (15 mL) was heated at 80 °C in the presence of a catalytic amount of HCl (conc) for 4 h. After cooling to room temperature the precipitate was filtered off, washed thoroughly with H_2_O (50 mL) and air-dried, affording **3** as dark-red crystalline powder. Yield: 97% (3.8 g), mp 135–137 °С; IR (KBr): 3419, 2915, 2848, 1698, 1662, 1619, 1464, 1364, 1334, 1102, 745 cm^−1^; ^1^H NMR (500 MHz, CDCl_3_/DMSO-*d*_6_ (9:1)) δ_H_ 0.74 (t, *J* = 7.0 Hz, 3H, CH_3_), 1.30–1.05 (m, 26H, 13CH_2_), 1.57 (q, *J* = 7.3 Hz, 2H, NCH_2_CH_2_), 3.63 (t, *J* = 7.3 Hz, 2H, NCH_2_), 6.66 (d, *J* = 7.6 Hz, 1H, H7), 6.70 (d, *J* = 7.6 Hz, 1H, H7’), 6.84 (ddd, *J* = 7.9 Hz, *J* = 7.8 Hz, *J* = 0.9 Hz, 1H, H5’), 6.88 (ddd, *J* = 7.9 Hz, *J* = 7.8 Hz, *J* = 0.9 Hz, 1H, H5), 7.14 (ddd, *J* = 7.8 Hz, *J* = 7.6 Hz, *J* = 0.9 Hz, 1H, H6’), 7.21 (ddd, *J* = 7.8 Hz, *J* = 7.6 Hz, *J* = 0.9 Hz, 1H, H6), 8.99 (d, *J* = 7.9 Hz, 1H, H4’), 9.02 (d, *J* = 7.9 Hz, 1H, H4), 9.81 (s, 1H, H1’); ^13^C{^1^H} NMR (125.7 MHz, CDCl_3_/DMSO-*d*_6_ (9:1)) δ_C_ 13.73 (CH_3_), 22.25 (C(15)H_2_), 26.63 (C(3)H_2_), 27.08 (NCH_2_CH_2_), 28.91 (C(4)H_2_+C(13)H_2_), 29.34–29.00 (C(5)H_2_–C(12)H_2_), 31.49 (C(14)H_2_), 39.66 (NCH_2_), 107.50 (C7), 109.30 (C7’), 121.30 (C5’), 121.32 (C3a), 121.64 (C5), 121.99 (C3a’), 129.30 (C4), 129.47 (C4’), 131.76 (C6), 132.12 (C6’), 132.62 (C3), 134.11 (C3’), 143.67 (C7a’), 144.22 (C7a), 167.50 (C2), 169.34 (C2’); ^15^N NMR (50.6 MHz, CDCl_3_/DMSO-*d*_6_ (9:1)) δ_N_ 136.2 (N1’), 142.5 (N1); Anal. calcd for C_32_H_42_N_2_O_2_: С, 78.97; Н, 8.70; N, 5.76; found: С, 78.85; Н, 8.57; N, 5.63.

The structure of **3** was established unambiguously by different of NMR correlation methods [80–81]. First, nOe's from NH^1'^ and H^1^-Alk let us to reveal H7' and H7, respectively (Supporting Information File 1). Then, structures of these moieties were determined by combination of ^1^H,^1^H COSY, ^1^H,^13^C HSQC/HMBC and ^1^H,^15^N HSQC/HMBC correlations. Almost perfect correlation between calculated [82] for a simplified model of **3** (with Alk = C_6_H_13_ instead of C_16_H_33_) and experimental ^13^C CS's (R^2^ = 0.998, Supporting Information File 1) strongly supports this structural hypothesis.

#### Preparation of solid isoindigo nanoparticles

The nanoprecipitation technique was used for preparation of nanoparticles based on amphiphilic isoindigo derivatives. The relevant derivative in different quantities was dissolved in DMF or THF (1 mL) at 60 °C, and the hot solution (0.5 mL) was added in 1 min to deionized water (25 mL) or phosphate buffer (25 mL) at 60 °C and stirred at 750 rpm. Nanoparticles were formed spontaneously. Solvent (THF) and part of water were removed under reduced pressure and the total volume was adjusted to 25 mL with deionized water.

### Methods

#### NMR spectroscopy

NMR experiments were performed with a 500 MHz (500 MHz for ^1^H NMR; 125 MHz for ^13^C NMR; 50.7 MHz for ^15^N NMR, respectively) spectrometer equipped with a 5 mm diameter gradient direct broad band probehead and a pulsed gradient unit capable of producing magnetic field pulse gradients in the *z*-direction of 53.5 G·cm^-1^. NMR experiments, carried out at 303 K. DPFGNOE [80], were obtained using a Hermite-shaped pulse for selective excitation. Chemical shifts (δ in ppm) are referenced to the solvent CDCl_3_ (δ = 7.27 ppm for ^1^H and 77.0 ppm for ^13^C NMR), to external CD_3_NO_2_ (380.2 ppm) for ^15^N NMR spectra (conversion factor to NH_3_: −380.2 ppm).

#### The quantum chemical calculations

The quantum chemical calculations were performed using Gaussian 03 software package [83]. Full geometry optimizations were carried out within the framework of DFT (B3LYP) method using 6-31G(d) basis sets. Chemical shifts (CSs) were calculated by the GIAO method at the same level of theory. All data were referred to TMS (^13^C) and NH_3_ (^15^N) chemical shifts, which were calculated under the same conditions.

#### Surface tension

Surface tension measurements were performed using the du Nouy ring detachment method (Kruss K6 Tensiometer, Hamburg, Germany). The experimental details are described elsewhere [84]. Briefly, the planar and spherical ring was placed parallel to the air–solvent interface. Between the surface tension analyses, the ring was cleaned by rinsing it with double-distilled water, followed by soaking it in nitric acid for 5–7 min, rinsing again with double-distilled water, and finally flame-drying. All glassware was soaked in nitric acid to avoid any contaminants, thoroughly rinsed with double-distilled water, and then steamed before use. Temperature was kept at 25 ± 0.2 °C during all experiments.

#### Solubilization study

The solubilization studies of the dye (Sudan I) were performed by adding an excess of crystalline Sudan I to the isoindigo derivatives solutions. These solutions were allowed to equilibrate for about 48 h at room temperature, followed by filtration, and the absorbance measured at 495 nm using the spectrophotometer Specord 250 Plus (Analytik Jena AG, Germany). Quartz cuvettes containing sample were used, with a 0.1 cm cell path.

For the solubilization studies of compounds **1**–**3**, saturated solutions were prepared in glass vessels by mixing of the excess of powdered compounds **1**–**3** with surfactants solutions with different concentrations. These solutions were allowed to equilibrate for about 48 h at room temperature, followed by filtration, and the absorbance of compounds **1**–**3** at appropriate wavelengths using the spectrophotometer Specord 250 Plus (Analytik Jena AG, Germany). Quartz cuvettes with a 1 cm cell path were used.

#### Dynamic light scattering

Dynamic light scattering (DLS) measurements were performed using the Malvern Instrument Zetasizer Nano (Worcestershire, UK). The measured autocorrelation functions were analysed by Malvern DTS software, applying the second-order cumulant expansion methods. The effective hydrodynamic radius (RH) was calculated according to the Einstein–Stokes equation: *D* = *k*_B_*T*/6πη*R*_H_, in which *D* is the diffusion coefficient, *k*_B_ is the Boltzmann constant, *T* is the absolute temperature, and η is the viscosity. The diffusion coefficient was measured at least in triplicate for each sample. The average error in these experiments was approximately 4%. The solutions were filtered with Millipore filters to remove dust particles from the scattering volume.

#### Fluorescence

Fluorescence spectra of pyrene (1 × 10^−6^ M) in water/DMF (50% v/v) solutions of **2a**–**h** were recorded at 25 °C on a Varian Cary Eclipse spectroﬂuorimeter (Varian, Inc., California, USA) with an excitation wavelength for pyrene at 335 nm using 1 cm path length quartz cuvettes. Emission spectra were recorded in the range of 350–500 nm.

#### Transmission electron microscopy (TEM)

Transmission electron microscopy (TEM) images were obtained using a microscope Hitachi HT7700, Japan. The images were acquired at an accelerating voltage of 110 kV. Samples were dispersed on 300 mesh copper grids with continuous carbon-formvar support films.

#### In vitro stability of SIPs

The SIP sample (5 mL) was poured into a dialysis bag that was immersed in 200 mL of phosphate buffer (pH 7.4) and incubated at 37 °C and 100 rpm. Bags with a pore size of 12 Da (Sigma-Aldrich) were used for this study. They were soaked in Milli-Q water for 12 h before use. After dialysis (3 h), a SIP sample (1 mL) of the fluid inside dialysis bag was withdrawn and assayed by determining the size and zeta potential using the Malvern Instrument Zetasizer Nano (Worcestershire, UK).

## Supporting Information

File 1 Additional experimental data.

## References

[1] Kar H, Gehrig D W, Laquai F, Ghosh S (2015). Nanoscale.

[2] Squillaci M A, Ferlauto L, Zagranyarski Y, Milita S, Müllen K, Samorì P (2015). Adv Mater.

[3] Jung E H, Bae S, Yoo T W, Jo W H (2014). Polym Chem.

[4] Yan Q, Luo Z, Cai K, Ma Y, Zhao D (2014). Chem Soc Rev.

[5] Ho C-C, Chen C-A, Chang C-Y, Darling S B, Su W-F (2014). J Mater Chem A.

[6] Jang B, Kwon H, Katila P, Lee S J, Lee H (2016). Adv Drug Delivery Rev.

[7] Khemthongcharoen N, Jolivot R, Rattanavarin S, Piyawattanametha W (2014). Adv Drug Delivery Rev.

[8] Walia S, Acharya A (2015). Beilstein J Nanotechnol.

[9] Viseu M I, Tatikolov A S, Correia R F, Costa S M B (2014). J Photochem Photobiol, A.

[10] Brinkmann J, Cavatorta E, Sankaran S, Schmidt B, van Weerd J, Jonkheijm P (2014). Chem Soc Rev.

[11] Xia Y, Peng L (2013). Chem Rev.

[12] Pakravan P, Kashanian S, Khodaei M M, Harding F J (2013). Pharmacol Rep.

[13] Ray D, Paul B K, Guchhait N (2013). J Photochem Photobiol, B.

[14] Prakash C, Raja S (2012). Mini-Rev Med Chem.

[15] Wee X K, Yang T, Go M L (2012). ChemMedChem.

[16] Marko D, Schätzle S, Friedel A, Genzlinger A, Zankl H, Meijer L, Eisenbrand G (2001). Br J Cancer.

[17] Moon M J, Lee S K, Lee J-W, Song W K, Kim S W, Kim J I, Cho C, Choi S J, Kim Y-C (2006). Bioorg Med Chem.

[18] Damiens E, Baratte B, Marie D, Eisenbrand G, Meijer L (2001). Oncogene.

[19] Myrianthopoulos V, Magiatis P, Ferandin Y, Skaltsounis A-L, Meijer L, Mikros E (2007). J Med Chem.

[20] Klein L L, Petukhova V, Wan B, Wang Y, Santasiero B D, Lankin D C, Pauli G F, Franzblau S G (2014). Bioorg Med Chem Lett.

[21] Bogdanov A V, Musin L I, Mironov V F (2015). ARKIVOC.

[22] Farag A A (2015). Drug Res (Stuttgart, Ger).

[23] Thanh N D, Giang N T K, Quyen T H, Huong D T, Toan V N (2016). Eur J Med Chem.

[24] Chohan Z H, Pervez H, Rauf A, Khan K M, Supuran C T (2004). J Enzyme Inhib Med Chem.

[25] Rane R A, Karunanidhi S, Jain K, Shaikh M, Hampannavar G, Karpoormath R (2016). Curr Top Med Chem.

[26] Medvedev A, Buneeva O, Glover V (2007). Biol: Targets Ther.

[27] Sriram D, Yogeeswari P, Meena K (2006). Pharmazie.

[28] Saleh A M, Al-As'ad R M, El-Abadelah M M, Sabri S S, Zahra J A, Alaskar A S, Aljada A (2014). Molecules.

[29] Saleh A M, El-Abadelah M M, Aziz M A, Taha M O, Nasr A, Rizvi S A A (2015). Cancer Lett.

[30] Xiao Z, Hao Y, Liu B, Qian L (2002). Leuk Lymphoma.

[31] Xiao Z, Qian L, Liu B, Hao Y (2000). Br J Haematol.

[32] Chen F, Li L, Ma D, Yan S, Sun J, Zhang M, Ji C, Hou M (2010). Leuk Res.

[33] Kashanian S, Khodaei M M, Pakravan P, Adibi H (2012). Mol Biol Rep.

[34] Kashanian S, Khodaei M M, Pakravan P (2010). DNA Cell Biol.

[35] Gusakov A V, Sinitsyn A P, Markov A V, Sinitsyna O A, Ankudimova N V, Berlin A G (2001). J Biotechnol.

[36] Kleeblatt D, Siyo B, Hein M, Iaroshenko V O, Iqbal J, Villinger A, Langer P (2013). Org Biomol Chem.

[37] Sassatelli M, Bouchikhi F, Messaoudi S, Anizon F, Debiton E, Barthomeuf C, Prudhomme M, Moreau P (2006). Eur J Med Chem.

[38] Ma W, Cheetham A G, Cui H (2016). Nano Today.

[39] Fumagalli G, Marucci C, Christodoulou M S, Stella B, Dosio F, Passarella D (2016). Drug Discovery Today.

[40] Luo C, Sun J, Sun B, He Z (2014). Trends Pharmacol Sci.

[41] Lin R, Cui H (2015). Curr Opin Chem Eng.

[42] Saad W S, Prud’homme R K (2016). Nano Today.

[43] Su H, Koo J M, Cui H (2015). J Controlled Release.

[44] Molla M R, Ghosh S (2014). Phys Chem Chem Phys.

[45] Hill J P, Shrestha L K, Ishihara S, Ji Q, Ariga K (2014). Molecules.

[46] Das A, Ghosh S (2016). Chem Commun.

[47] Sikder A, Das A, Ghosh S (2015). Angew Chem, Int Ed.

[48] Shankar B H, Jayaram D T, Ramaiah D (2015). Chem – Eur J.

[49] Lock L L, LaComb M, Schwarz K, Cheetham A G, Lin Y-a, Zhang P, Cui H (2013). Faraday Discuss.

[50] Bogdanov A V, Pashirova T N, Musin L I, Krivolapov D B, Zakharova L Y, Mironov V F, Konovalov A I (2014). Chem Phys Lett.

[51] Soussan E, Cassel S, Blanzat M, Rico-Lattes I (2009). Angew Chem, Int Ed.

[52] Kumar A, Chen F, Mozhi A, Zhang X, Zhao Y, Xue X, Hao Y, Zhang X, Wang P C, Liang X-J (2013). Nanoscale.

[53] Verma G, Hassan P A (2013). Phys Chem Chem Phys.

[54] Liang Y, Deng X, Zhang L, Peng X, Gao W, Cao J, Gu Z, He B (2015). Biomaterials.

[55] Karnik R, Gu F, Basto P, Cannizzaro C, Dean L, Kyei-Manu W, Langer R, Farokhzad O C (2008). Nano Lett.

[56] Mora-Huertas C E, Fessi H, Elaissari A (2010). Int J Pharm.

[57] Bilati U, Allémann E, Doelker E (2005). Eur J Pharm Sci.

[58] Zhu Z (2013). Biomaterials.

[59] Tong R, Yala L, Fan T M, Cheng J (2010). Biomaterials.

[60] Fessi H, Puisieux F, Devissaguet J P, Ammoury N, Benita S (1989). Int J Pharm.

[61] Quintanar-Guerrero D, Allémann E, Fessi H, Doelker E (1998). Drug Dev Ind Pharm.

[62] Lakkakula J R, Maçedo Krause R W (2014). Nanomedicine (London, U K).

[63] Montasser I, Shahgaldian P, Perret F, Coleman A W (2013). Int J Mol Sci.

[64] Ansell S M, Johnstone S A, Tardi P G, Lo L, Xie S, Shu Y, Harasym T O, Harasym N L, Williams L, Bermudes D (2008). J Med Chem.

[65] Geng Y, Romsted L S, Menger F (2006). J Am Chem Soc.

[66] Tanford C (1972). J Phys Chem.

[67] Tehrani-Bagha A R, Holmberg K (2013). Materials.

[68] Aguiar J, Carpena P, Molina-Bolívar J A, Ruiz C C (2003). J Colloid Interface Sci.

[69] Yan M, Li B, Zhao X (2010). Food Chem.

[70] Gabdrakhmanov D R, Valeeva F G, Syakaev V V, Lukashenko S S, Zakharov S V, Kuryashov D A, Bashkirtseva N Y, Zakharova L Y, Latypov S K, Sinyashin O G (2015). Mendeleev Commun.

[71] Ren Y, Hiszpanski A M, Whittaker-Brooks L, Loo Y-L (2014). ACS Appl Mater Interfaces.

[72] Williams H D, Trevaskis N L, Charman S A, Shanker R M, Charman W N, Pouton C W, Porter C J H (2013). Pharmacol Rev.

[73] Reddy L H, Bazile D (2014). Adv Drug Delivery Rev.

[74] Mahajan S, Mahajan R K (2013). Adv Colloid Interface Sci.

[75] Noori S, Naqvi A Z, Ansari W H, Kabir-ud-Din (2014). Colloids Surf, B.

[76] Bhattacharya S, Biswas J (2010). Langmuir.

[77] Sun Z, Nicolosi V, Rickard D, Bergin S D, Aherne D, Coleman J N (2008). J Phys Chem C.

[78] Gharibi H, Moosavi-Movahedi Z, Javadian S, Nazari K, Moosavi-Movahedi A A (2011). J Phys Chem B.

[79] Gordon A, Ford R (1972). The Chemist’s Companion. A Handbook of Practical Data, Techniques, and Reference.

[80] Derome A E (1988). Modern NMR Techniques for Chemistry Research.

[81] Stott K, Stonehouse J, Keeler J, Hwang T-L, Shaka A J (1995). J Am Chem Soc.

[82] Latypov S, Balandina A, Boccalini M, Matteucci A, Usachev K, Chimichi S (2008). Eur J Org Chem.

[83] (2003). Gaussian 03.

[84] Pashirova T N, Lukashenko S S, Zakharov S V, Voloshina A D, Zhiltsova E P, Zobov V V, Souto E B, Zakharova L Y (2015). Colloids Surf, B.

