# Supplementary material for: Nanoscale isoindigo-carriers: self-assembly and tunable properties
Source: Beilstein J Nanotechnol. 2017 Feb 1;8:313–24. doi: 10.3762/bjnano.8.34 (PMC5301918; doi:10.3762/bjnano.8.34)
Supplement: File 1 — Additional experimental data. [file Beilstein_J_Nanotechnol-08-313-s001.pdf]

Supporting Information

for

Nanoscale isoindigo-carriers: self-assembly and tunable properties

Tatiana N. Pashirova*, Andrei V. Bogdanov, Lenar I. Musin, Julia K. Voronina, Irek R. Nizameev, Marsil K. Kadirov, Vladimir F. Mironov, Lucia Ya. Zakharova, Shamil K. Latypov and Oleg G. Sinyashin

Address: A.E. Arbuzov Institute of Organic and Physical Chemistry, Kazan Scientific Center, Russian Academy of Sciences, Arbuzov str. 8, Kazan, 420088, Russian Federation

Email: Tatiana Pashirova* - tatyana_pashirova@mail.ru

*Corresponding author

Additional experimental data

**Contents**	**Pages**
General remarks	S3
Solution preparation procedure	S3
Table S1. The distribution of sizes of **2b**, **2d**, **2e**, **2f**, **2h** particles in water	S5
Figure S1. Zeta-potential of 2d, 2e, 2f and 2h particles	S6
Figure S2. Analysis of the size distribution of **2d** after incubation in phosphate buffer	S7
Table S2. Analysis of the size distribution (diameter, nm) of **2c–g** particles	S8
Figure S3. Analysis of the size distribution of **2c–g** particles	S9
Figure S4. Analysis of the size distribution of **2c–g** particles	S10
Figure S5. Zeta-potential of **2h** particles	S11
Figure S6. Analysis of the size distribution of **2g** in phosphate buffer /DMF	S12
Figure S7. Analysis of the size distribution of **2h** particles	S13
Figure S8. The histogram of the head fragment surface distribution	S14
Table S3. The packing parameter, *P*	S15
Figure S9. Surface tension isotherms of **2e**, **2g**, **2h**	S16
Table S4 Cmc and solubilization capacity by the method of solubilization of Sudan I	S17
Figure S10. Dependence of the intensity ratio (I_0/I) for pyrene	S18
Figure S11–S19. Absorption profile of **2a, 2c–h, 3, 1g** in chloroform	S19-S21
Figure S20. Absorption profile of **1g** in ethanol	S22
Figure S21. Absorption profile of **1g** in DMF	S23
Table S5. Molar extinction coefficient of **1g**, **2a**, **2c**, **2d**, **2e-h** and **3**.	S24
Figure S22. Absorption profile of **2a**, **1g** and **3** in chloroform and water–SDS solutions	S25
Figure S23. Absorption profile of the **3** in water–micellar solutions	S26
Figure S24. Absorbance of the **3** at λ_{max} = 300 nm in water–micellar solutions	S27
Figure S25. 1D ^{1}H, ^{13}C DEPT and ^{13}C{^{1}H} NMR spectra of **3**	S28
Figure S26. 2D ^{1}H,^{1}H COSY NMR spectra of **3**	S29
Figure S27. 2D ^{1}H,^{13}C HSQC NMR spectra of **3**	S30
Figure S28. 2D ^{1}H,^{13}C HMBC NMR spectra of **3**	S31
Figure S29. 1D ^{1}H and ^{1}H DPFGNOE NMR spectra of **3**	S32
Figure S30. 2D ^{1}H,^{15}N HSQC NMR spectra of **3**	S33
Figure S31. 2D ^{1}H,^{15}N HMBC NMR spectra of **3**	S34
Table S6. Calculated (B3LYP/6-31G(d)//B3LYP/6-31G(d)) for **3’** and experimental for **3** ^{13}C and ^{15}N (in $CDCl_3$/DMSO-d_6 (9:1)) CSs	S35-36
Figure S32. Correlations of calculated (B3LYP/6-31G(d)//B3LYP/6-31G(d), for **3’**) versus experimental ^{13}C chemical shifts for **3**	S37
References	S38

General remarks

All the manipulations were performed in open vessels. NMR experiments were performed with a 500 MHz (500 MHz for ^{1}H NMR; 125 MHz for ^{13}C NMR; 50.7 MHz for ^{15}N NMR, respectively) spectrometer equipped with a 5 mm diameter gradient direct broad band probehead and a pulsed gradient unit capable of producing magnetic field pulse gradients in the *z*-direction of 53.5 G·cm^{-1}. NMR experiments were carried out at 303 K. DPFGNOE were obtained using a Hermite-shaped pulse for selective excitation. Chemical shifts (δ in ppm) are referenced to the solvent $CDCl_3$ (δ = 7.27 ppm for ^{1}H and 77.0 ppm for ^{13}C NMR), to external CD_3NO_2 (380.2 ppm) for ^{15}N NMR spectra (conversion factor to NH_3: −380.2 ppm).

The quantum chemical calculations were performed using Gaussian 03 software package. Full geometry optimizations have been carried out within the framework of DFT (B3LYP) method using 6-31G(d) basis sets. Chemical shifts (CSs) were calculated by the GIAO method at the same level of theory**.** All data were referred to TMS (^{13}C) and NH_3 (^{15}N) chemical shifts, which were calculated under the same conditions. IR spectra were measured with Bruker Vector-22 spectrometer. Melting points were measured with a Stuart digital SMP10 apparatus and uncorrected. Elemental analyses for C, H and N were performed using a EuroVector 2000 CHNS-3 analyzer, Italy.

Solution preparation procedure

Compounds **2a–h** were dissolved in DMF at 60 °C followed by preheated (60 °C) water addition to form 1:1 mixture with desired concentration. Purified water (18.2 MΩ·cm resistivity at 25 ºC) from Direct-Q 5 UV equipment was used for all solution preparation.

Table S1: The distribution of sizes (diameter, nm), polydispersity index values (PdI) and Zeta Potential of **2b**, **2d**, **2e**, **2f, 2h** particles in water, 25 °C.

Mean ± standard deviation from three independent samples,

Dye	Dye concentration, wt %	Diameter, nm		PdI	Zeta Potential, mV	Diameter, nm		PdI	Diameter, nm		PdI
		Intensity, %	Number, %			Intensity, %	Number, %		Intensity, %	Number, %	
		storage time: 1st day				storage time: 12th day			storage time: 80th day		
**2b***	0.02	712±148	712±148	0.15±0.02	-	not stable	not stable		not stable	not stable	
**2d***	0.02	164±28	106±21	0.08±0.02	-34.1±0.5	164±26	91±19	0.1±0.01	164±27	106±22	0.08±0.01
**2d****	0.02	190±25	122±20	0.28±0.04	-	-	-	-	-	-	-
**2d***	0.05	255±28	142±23	0.27±0.03	-40±2	-	-	-	-	-	-
**2d***	0.1	1484±20	1282±20	0.25±0.03	-18.8±0.5	-	-	-	-	-	-
**2e***	0.02	164±29	106±22	0.07±0.02	-27.8±0.7	-	-	-	-	-	-
**2f***	0.02	220±33	122±22	0.15±0.02	-30.1±0.7	190±31	142±27	0.17±0.01	220±35	142±25	0.12±0.03
**2h***	0.02	255±35	142±22	0.14±0.01	-38.6±0.8	255±36	164±27	0.14±0.02	255±45	190±35	0.11±0.02
**2h*****	0.02	342±64	255±47	0.33±0.04	-	295±33	190±25	0.25±0.03	342±68	295±59	0.25±0.05

* using organic solvent DMF
** synthesis in phosphate buffer
*** using organic solvent THF

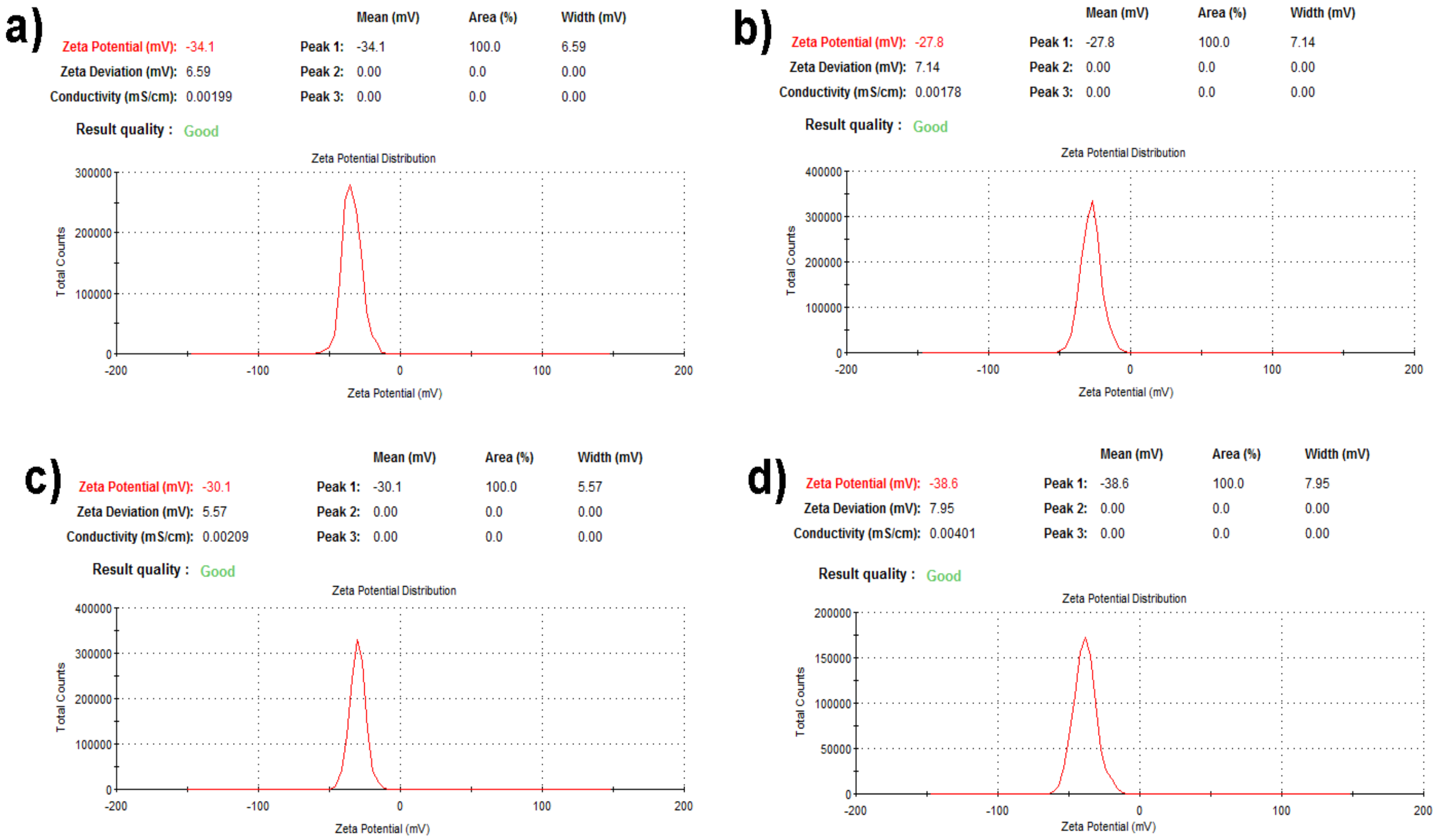

Figure S1: Zeta potential (mV) of **2d** (a), **2e** (b), **2f** (c) and **2h** (d) particles in water, 25 °C.

			Diam. (nm)	% Number	Width (nm)
Z-Average (d.nm):	871.4	Peak 1:	157.5	56.9	52.06
PdI:	0.443	Peak 2:	698.5	43.1	370.6
Intercept:	0.883	Peak 3:	0.000	0.0	0.000

Result quality : Good

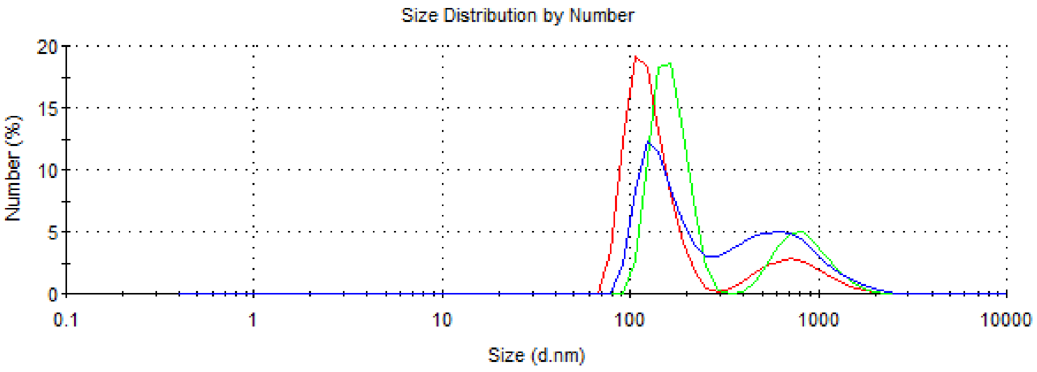

Figure S2: Analysis of the size distribution of **2d** particles after incubation in phosphate buffer, pH 7.4, 37 °C.

Table S2: The distribution of sizes (diameter, nm) of **2c–g** particles in water/DMF (50% v/v) solutions using the number parameter and polydispersity index values (PdI) of the system, 25 °C. Mean ± standard deviation from three independent samples.

Concentration (mM)	**2c**		**2d**		**2e**		**2f**		**2g**	
	Diameter (nm)	PdI	Diameter (nm)	PdI	Diameter (nm)	PdI	Diameter (nm)	PdI	Diameter (nm)	PdI
0.01			-	-	-	-	-	-	142±35	0.06±0.02
0.02			59±10; 220±25	0.14±0.03	164±42	0.05±0.03	-	-	106±35	0.06±0.02
0.04			-	-	-	-	-	-	106±30	0.06±0.03
0.05			68±3; 164±34	0.08±0.03	164±50	0.024±0.01	-	-	106±32	0.05±0.03
0.08			-	-	-	-	-	-	142±38	0.05±0.02
0.1			164±39	0.085±0.02	164±47	0.032±0.02	-	-	106±35	0.04±0.02
0.2			164±37	0.056±0.02	142±38	0.055±0.03	164±44	0.05±0.03	92±21	0.08±0.03
0.4	396±95	0.082±0.02	-	-	-	-	-	-	190±46	0.02±0.01
0.5			295±86	0.105±0.04	190±67	0.06±0.02	164±40	0.07±0.04	123±28	0.045±0.02
0.6	342±89	0.07±0.05	-	-	-	-	-	-	123±27	0.06±0.03
1	712±131	0.04±0.01	190±65	0.04±0.04	164±61	0.03±0.02	190±49	0.13±0.05	123±27	0.05±0.02
2			342±54	0.154±0.02	-	-	460±150	0.04±0.02	240±64	0.21±0.1

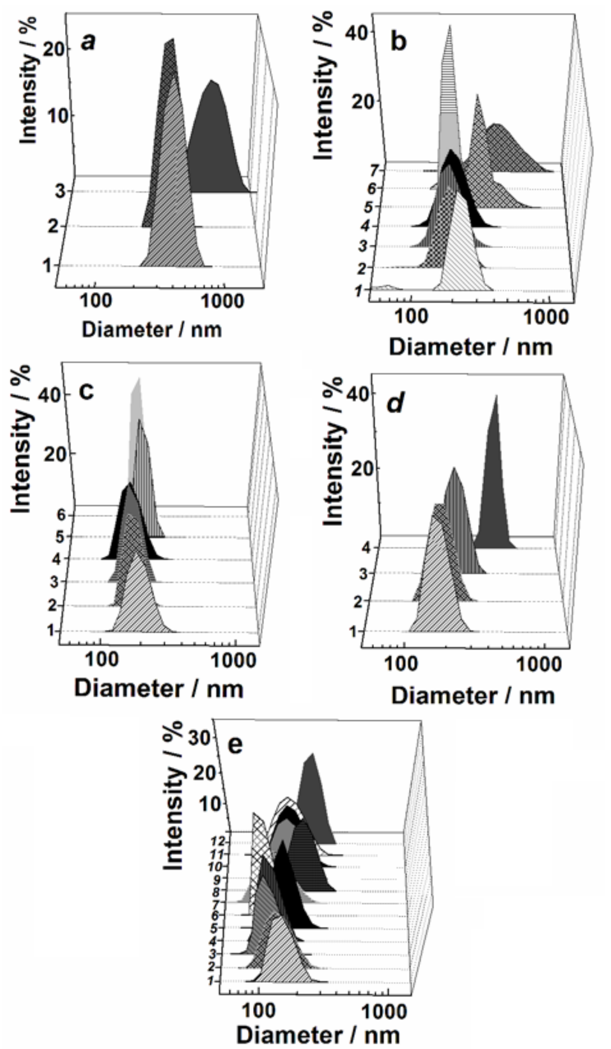

Figure S3: Analysis of the size distribution of **2c–g** particles in water/DMF (50% v/v) solutions using the intensity parameter, 25 °C, a - $C_{\mathbf{2c}}$, mM: 0.4 (1); 0.6 (2); 1 (3); b - $C_{\mathbf{2d}}$, mM: 0.02 (1); 0.05 (2); 0.1 (3); 0.2 (4); 0.5 (5); 1 (6); 2 (7); c - $C_{\mathbf{2e}}$, mM: 0.02 (1); 0.05 (2); 0.1 (3); 0.2 (4); 0.5 (5); 1 (6); d - $C_{\mathbf{2f}}$, mM: 0.2 (1); 0.5 (2); 1 (3); 2 (4); e - $C_{\mathbf{2g}}$, mM: 0.01 (1); 0.02 (2); 0.04 (3); 0.05 (4); 0.08 (5); 0.1 (6); 0.2 (7); 0.4 (8); 0.5 (9); 0.6 (10); 1 (11); 2 (12).

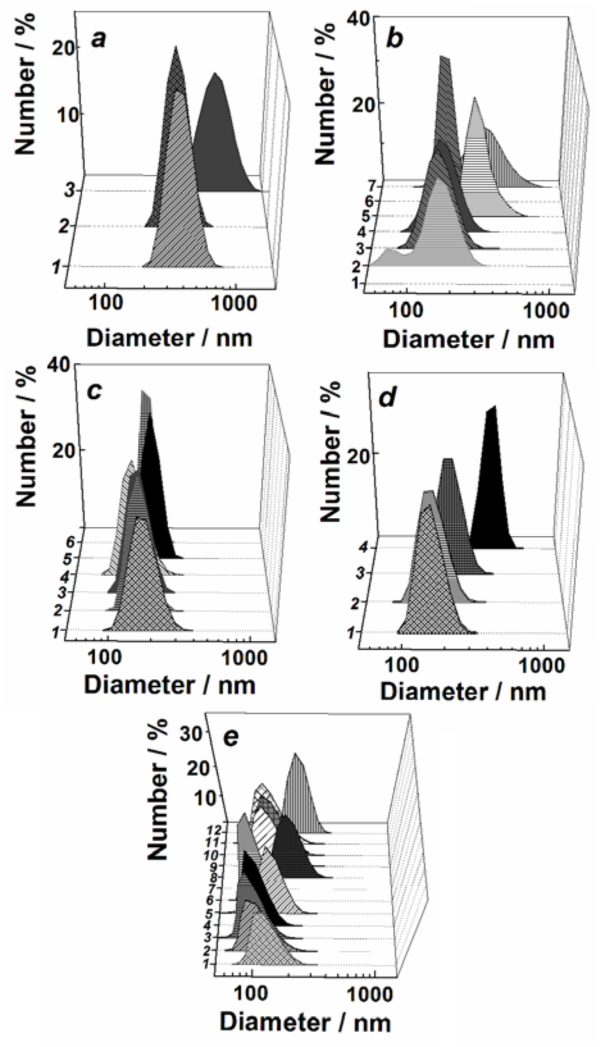

Figure S4: Analysis of the size distribution of **2c–g** particles in water/DMF (50% v/v) solutions using the number parameter, 25 °C, (a) - C_{2c}, mM: 0.4 (1); 0.6 (2); 1 (3); (b) - C_{2d}, mM: 0.02 (1); 0.05 (2); 0.1 (3); 0.2 (4); 0.5 (5); 1 (6); 2 (7); (c) - C_{2e}, mM: 0.02 (1); 0.05 (2); 0.1 (3); 0.2 (4); 0.5 (5); 1 (6); (d) - C_{2f}, mM: 0.2 (1); 0.5 (2); 1 (3); 2 (4); (e) - C_{2g}, mM: 0.01 (1); 0.02 (2); 0.04 (3); 0.05 (4); 0.08 (5); 0.1 (6); 0.2 (7); 0.4 (8); 0.5 (9); 0.6 (10); 1 (11); 2 (12).

			Mean (mV)	Area (%)	Width (mV)
Zeta Potential (mV):	-30.6	Peak 1:	-30.6	100.0	9.89
Zeta Deviation (mV):	9.89	Peak 2:	0.00	0.0	0.00
Conductivity (mS/cm):	0.0177	Peak 3:	0.00	0.0	0.00

Result quality : Good

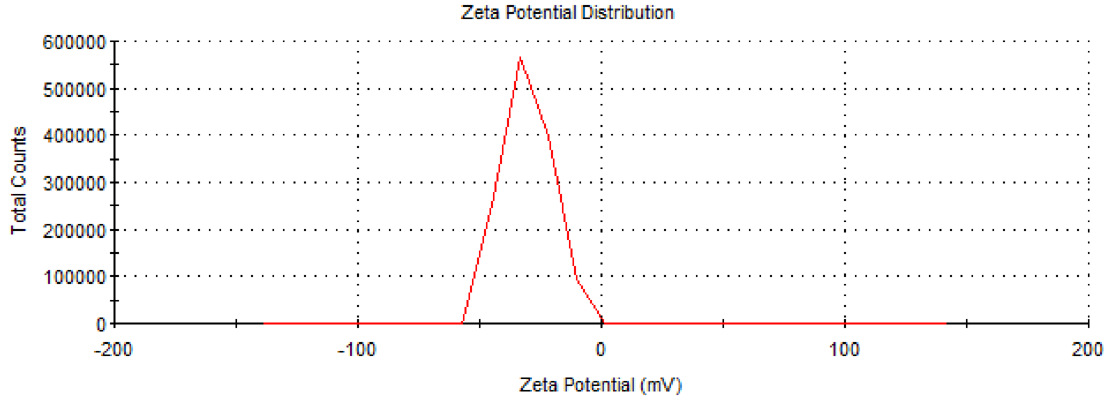

Figure S5: Zeta potential (mV) of **2h** particles in water/DMF (50% v/v), 25 °C, $C_{\mathbf{2h}}$, mM: 0.2.

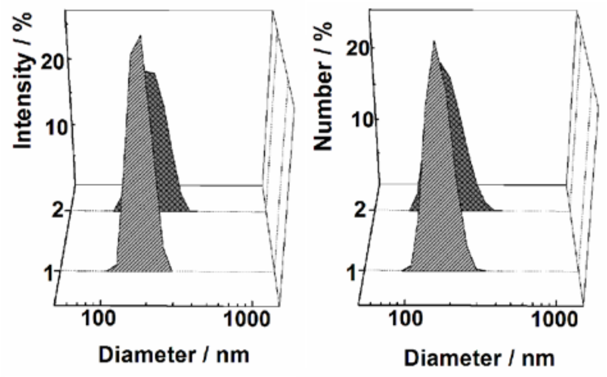

Figure S6: Analysis of the size distribution of **2h** particles in water/DMF (50% v/v) solutions using the intensity and number parameters, 50 °C, $C_{\mathbf{2h}}$, mM: 0.4 (1); 0.6 (2).

			Diam. (nm)	% Number	Width (nm)
Z-Average (d.nm):	437.2	Peak 1:	414.8	100.0	121.7
PdI:	0.046	Peak 2:	0.000	0.0	0.000
Intercept:	0.934	Peak 3:	0.000	0.0	0.000

Result quality : Good

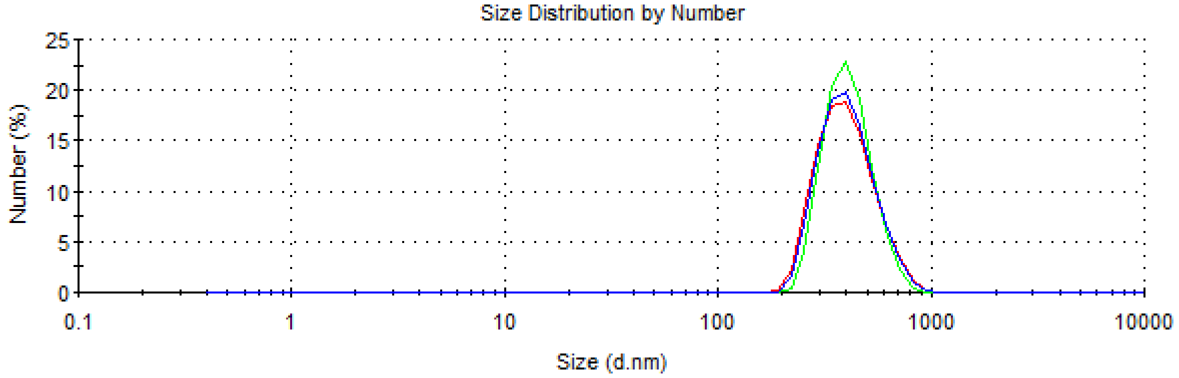

Figure S7: Analysis of the size distribution of **2g** in phosphate buffer/DMF (50% v/v) solutions using number parameters, 25 °C, $C_{\mathbf{2g}}$, mM: 0.6.

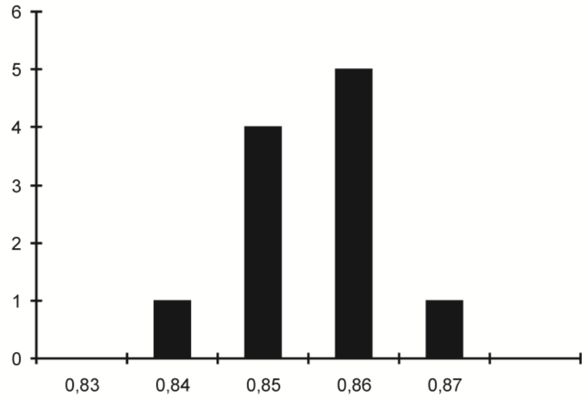

Figure S8: The histogram of the head fragment surface distribution in the isoindigo derivatives for which the data are present in the Cambridge Structural Database.

Table S3: The volume of the hydrophobic spacer, v_0, the polar head surface area, a, the chain length of the hydrophobic fragment, l and the packing parameter, P.

dye	l, nm	v_0, nm^3	a, nm^2	P
**1g**	2.17	0.46	0.86[a]	0.25
**2a**	0.28	0.11	0.86	0.46
**2b**	0.91	0.38	0.86	0.49
**2c**	1.16	0.49	0.86[b]	0.5
**2d**	1.41	0.6	0.85[c]	0.5
**2e**	1.67	0.7	0.86	0.49
**2f**	1.92	0.81	0.86	0.49
**2g**	2.17	0.92	0.86	0.49
**2h**	2.42	1.02	0.86	0.49
**3**	2.17	0.46	1.72	0.12

[a] reference [2]

[b] reference [3]

[c] reference [4]

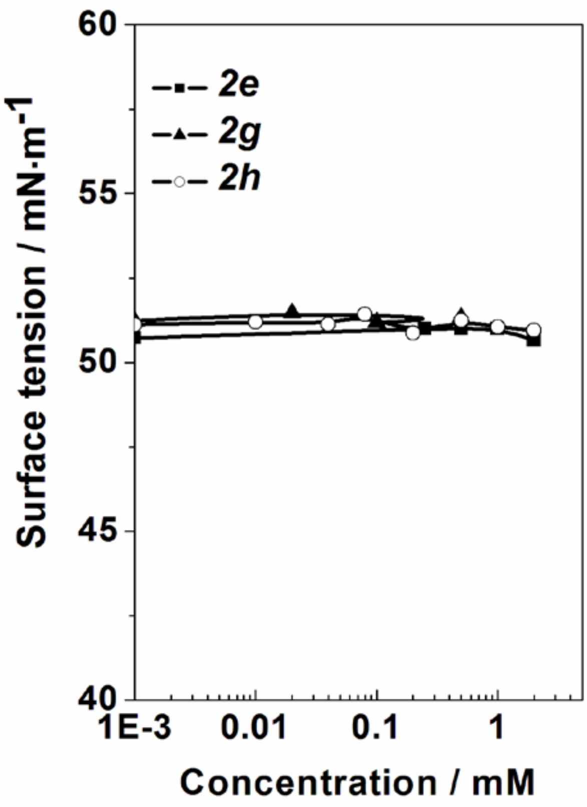

Figure S9: Surface tension isotherms of **2e**, **2g**, **2h** in water/DMF (50% v/v) solution; 25 °C.

Table S4: CMC values and solubilization capacity, determined by the method of solubilization of Sudan I; 25 °C

Compound	CMC × 10^3, M	Solubilization capacity
**2g**	0.2	0.4
**2h**	0.1	0.52
SDS	10	0.027
CTAB	0.98[b]	0.189

[a]CMC value determined by fluorimetry
[b]from [5] at 30 °C.

Solubilization capacity (*S*) is the parameter of system, which quantitively characterizes its ability to solubilize (bind) organic substrate. According to the published data, *S* value, that is, number of moles of dye solubilized by one mole micellized surfactant, can be calculated by Equation S1 [6]:

$$S = B / \varepsilon L \quad , \qquad \text{(S1)}$$

where *B* is the parameter of slope (slope of the dependence of absorbance of dye vs surfactant concentration above CMC), ε is the extinction coefficient of dye (ε = 8700 $M^{-1}\cdot cm^{-1}$), and *L* is the length of the optical path.

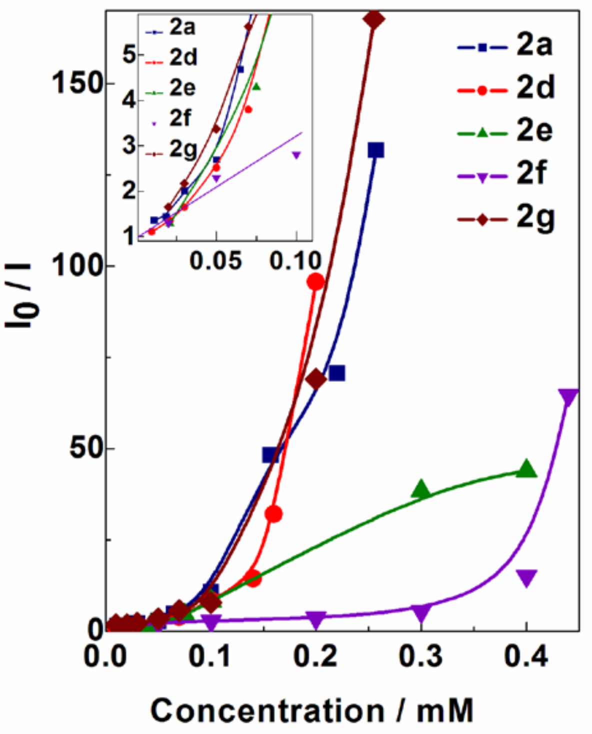

Figure S10: Dependence of the intensity ratio (I_0/I) for pyrene in the absence (I_0) and in the presence of the **2a**, **2d–2g** (I) on their concentration in water/DMF (50% v/v) solutions, 25 °C.

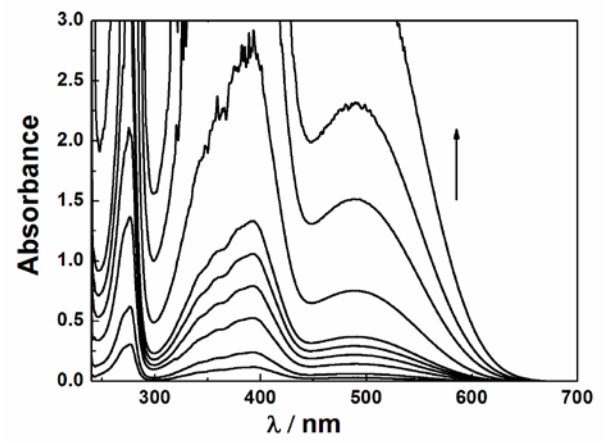

Figure S11: Absorption profile of **2a** in chloroform, $C_{\mathbf{2a}}$ (mM): 0.01; 0.02; 0.04; 0.06; 0.08; 0.1; 0.2; 0.4; 0.6; 1, L = 1 cm.

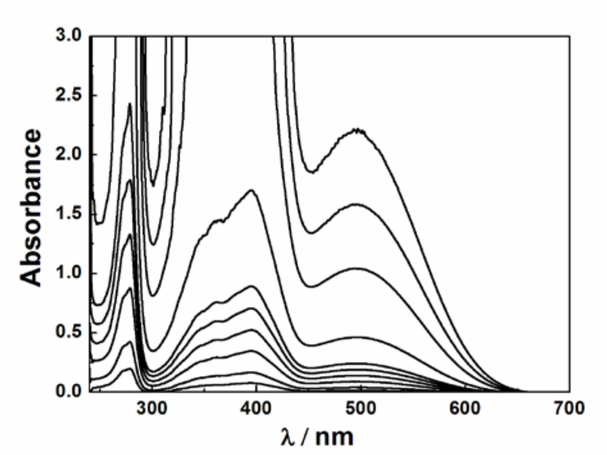

Figure S12: Absorption profile of **2c** in chloroform, $C_{\mathbf{2c}}$ (mM): 0.01; 0.02; 0.04; 0.06; 0.08; 0.1; 0.2; 0.4; 0.6; 0.8 mM, L = 1 cm.

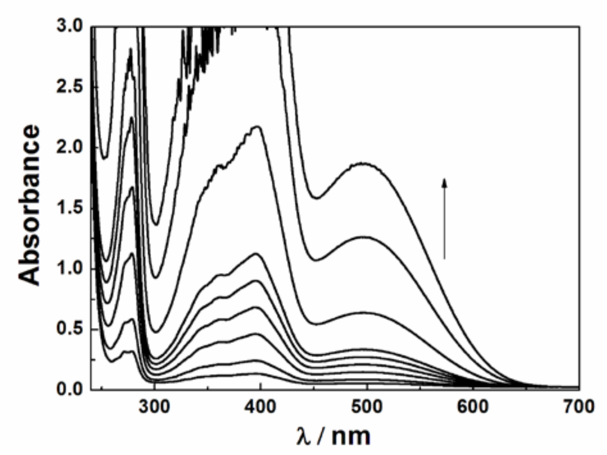

Figure S13: Absorption profile of **2d** in chloroform, $C_{\mathbf{2d}}$ (mM): 0.01; 0.02; 0.04; 0.06; 0.08; 0.1; 0.2; 0.4; 0.6 mM, L = 1cm.

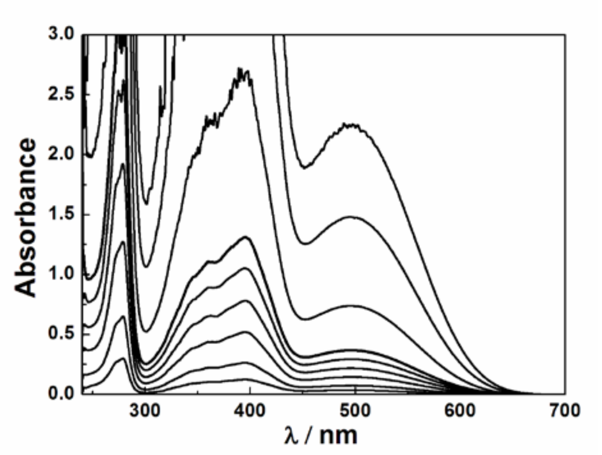

Figure S14: Absorption profile of **2e** in chloroform, $C_{\mathbf{2e}}$ (mM): 0.01; 0.02; 0.04; 0.06; 0.08; 0.1; 0.2; 0.4; 0.6 mM, L = 1 cm.

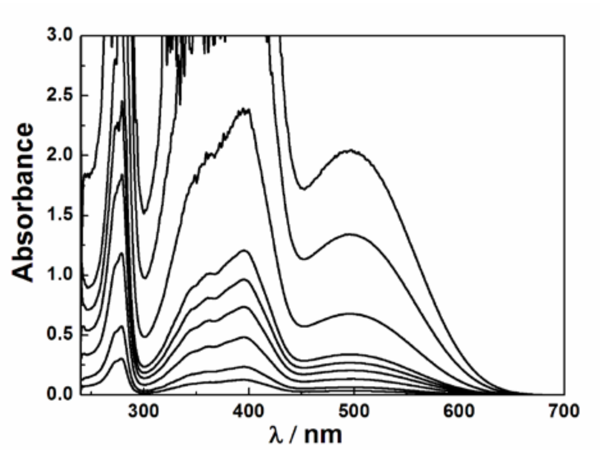

Figure S15: Absorption profile of **2f** in chloroform, $C_{\mathbf{2f}}$ (mM): 0.01; 0.02; 0.04; 0.06; 0.08; 0.1; 0.2; 0.4; 0.6 mM, L = 1 cm.

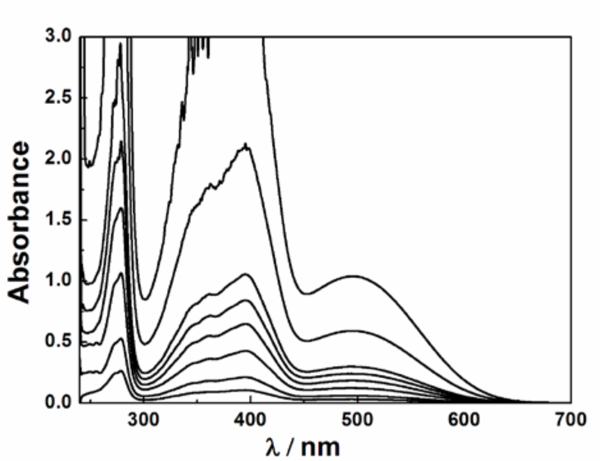

Figure S16: Absorption profile of **2g** in chloroform, $C_{\mathbf{2g}}$ (mM): 0.01; 0.02; 0.04; 0.06; 0.08; 0.1; 0.2; 0.3; L = 1 cm.

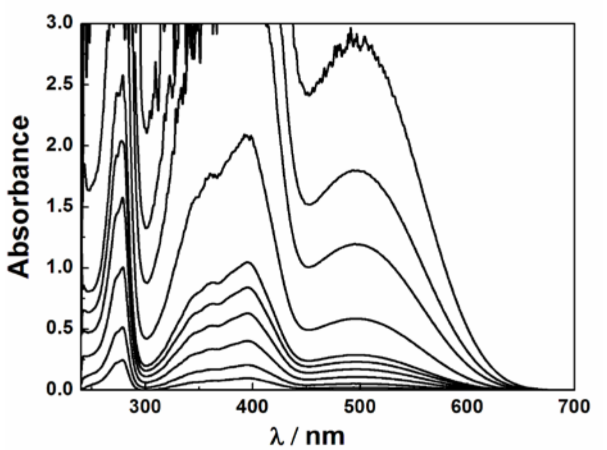

Figure S17: Absorption profile of **2h** in chloroform, $C_{\mathbf{2h}}$ (mM): 0.01; 0.02; 0.04; 0.06; 0.08; 0.1; 0.2; 0.4; 0.6; 1, L = 1 cm.

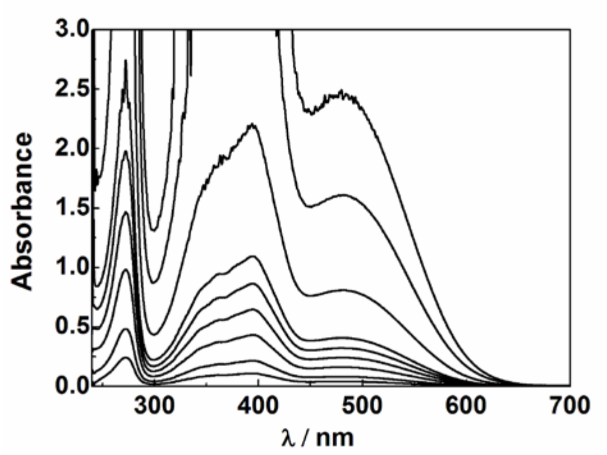

Figure S18: Absorption profile of **3** in chloroform, $C_{\mathbf{3}}$ (mM): 0.01; 0.02; 0.04; 0.06; 0.08; 0.1; 0.2; 0.4; 0.6, L = 1 cm.

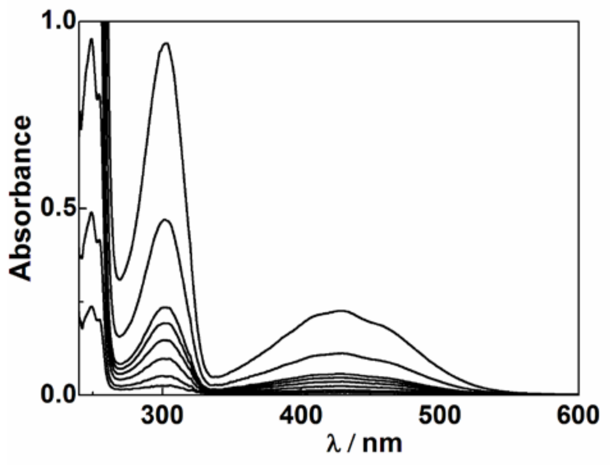

Figure S19: Absorption profile of **1g** in chloroform, $C_{\mathbf{1g}}$ (mM): 0.0125; 0.025; 0.05; 0.075; 0.1; 0.125; 0.25; 0.5, L = 1 cm.

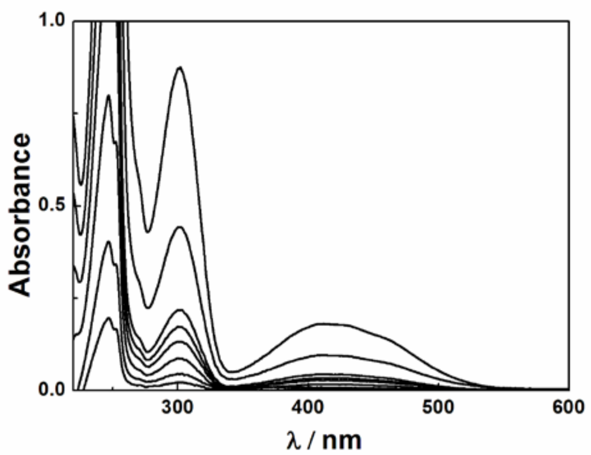

Figure S20: Absorption profile of **1g** in ethanol, $C_{\mathbf{1g}}$ (mM): 0.0125; 0.025; 0.05; 0.075; 0.1; 0.125; 0.25; 0.5, L = 1 cm.

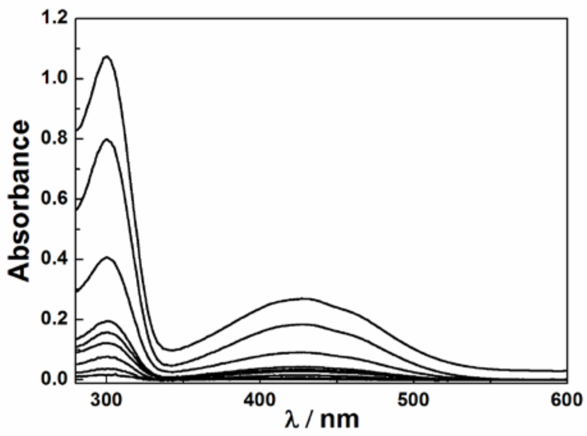

Figure S21: Absorption profile of **1g** in DMF, $C_{\mathbf{1g}}$ (mM): 0.01; 0.02; 0.04; 0.06; 0.08; 0.1; 0.2; 0.4; 1, L = 1 cm.

Table S5: Molar extinction coefficient (ε) of **1g**, **2a**, **2c**, **2d**, **2e–h** and **3**.

Dye	λ_{max}/nm/ (ε/M^{-1} cm^{-1})	λ_{max}/nm/ (ε/M^{-1} cm^{-1})	λ_{max}/nm/ (ε/M^{-1} cm^{-1})
**1g**[a]	412 (366)	300 (1749)	247 (15783)
**1g**[b]	428 (466)	300 (2012)	-
**1g**[c]	428 (447)	300 (1864)	248 (18897)
**2a**	490 (3866)	393 (13447)	275 (35871)
**2c**	495 (2647)	395 (8597)	278 (22419)
**2d**	495 (3083)	395 (10775)	278 (27882)
**2e**	495 (3736)	395 (13156)	278 (32211)
**2f**	495 (3384)	395 (11946)	278 (29294)
**2g**	495 (2952)	395 (10761)	278 (26773)
**2h**	495 (2936)	395 (10409)	278 (25451)
**3**	480 (4167)	395 (10828)	272 (24329)

[a]EtOH

[b]DMF

[c]$CHCl_3$

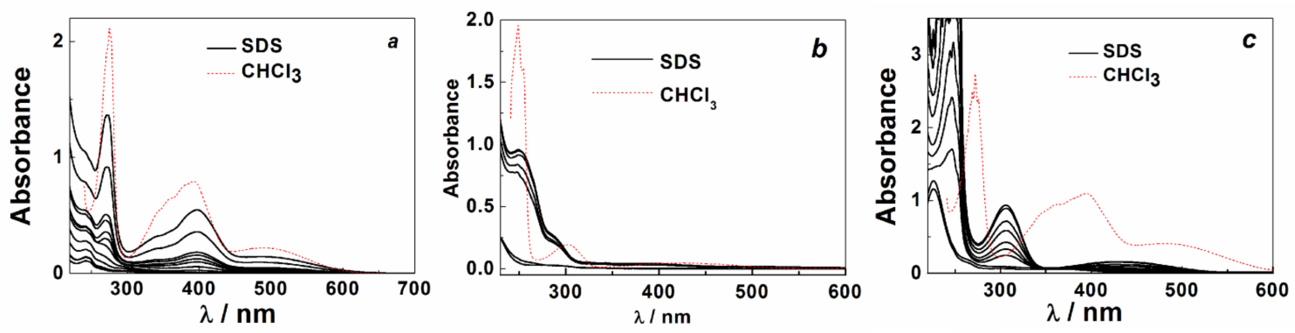

Figure S22: Absorption profile of **2a** (a), **1g** (b) and **3** (c) in chloroform and water–SDS solutions in the presence of increasing SDS concentration, 25 °C, (a): C_{SDS} = 1–60 mM, (b): C_{SDS} = 2–200 mM, (c): C_{SDS} = 2–200 mM.

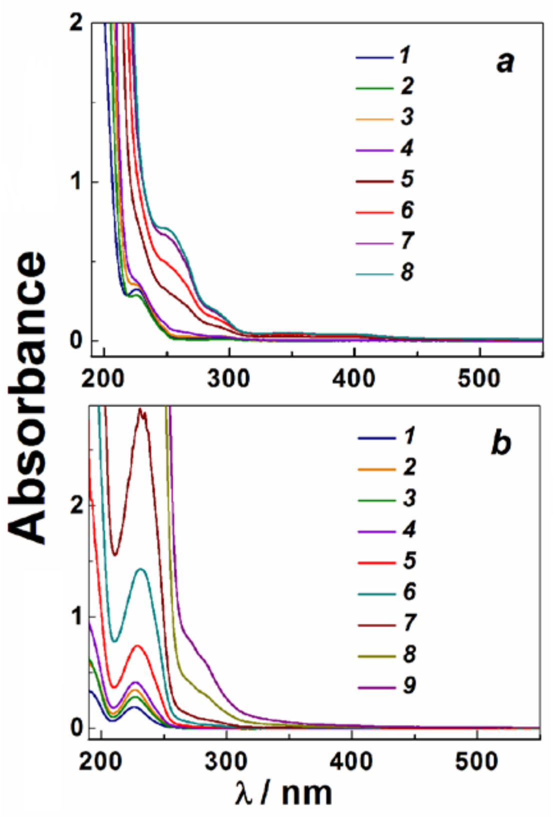

Figure S23: Absorption profile of the **3** in water–micellar solutions in the presence of increasing concentration of the CTAB (a) and Tween 80 (b), 25 °C, C_{CTAB}, mM: 0.2 (1); 0.4 (2); 0.6 (3); 0.8 (4); 2 (5); 5 (6); 10 (7); 20 (8), $C_{\text{Tween 80}}$, mM: 0.02 (1); 0.04 (2); 0.06 (3); 0.08 (4); 0.2 (5); 0.5 (6); 0.6 (7); 1 (8); 5 (9).

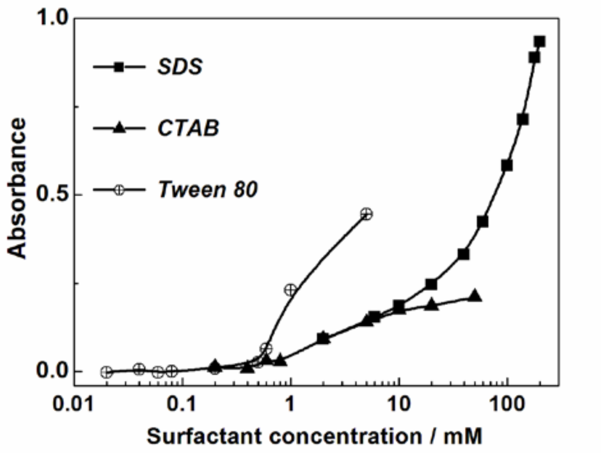

Figure S24: Absorbance of the **3** at λ_{max} = 300 nm in aqueous micellar surfactants solution, 25 °C; optical length 1 cm.

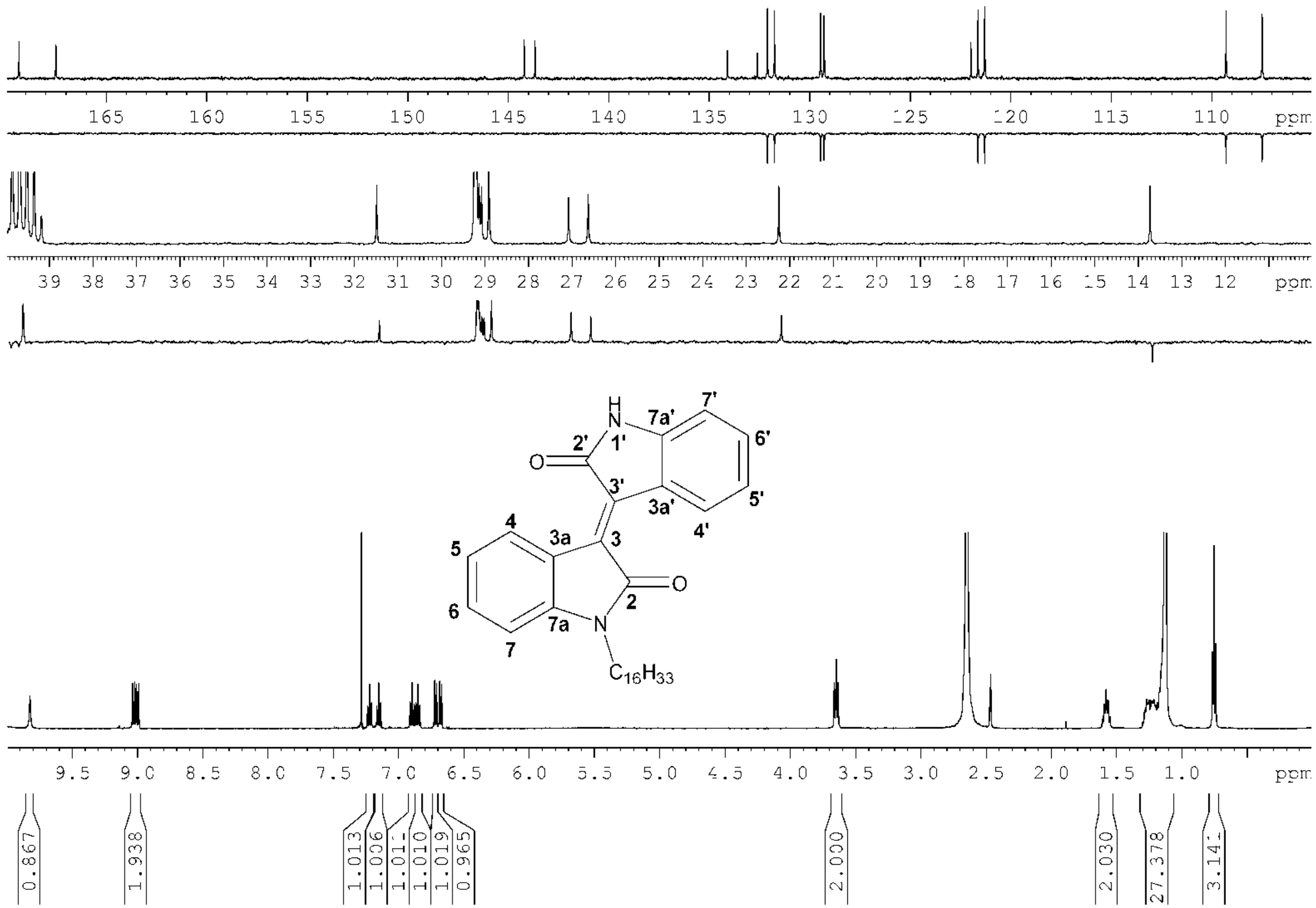

Figure S25: 1D ^{1}H, ^{13}C DEPT and ^{13}C{^{1}H} NMR spectra of **3**.

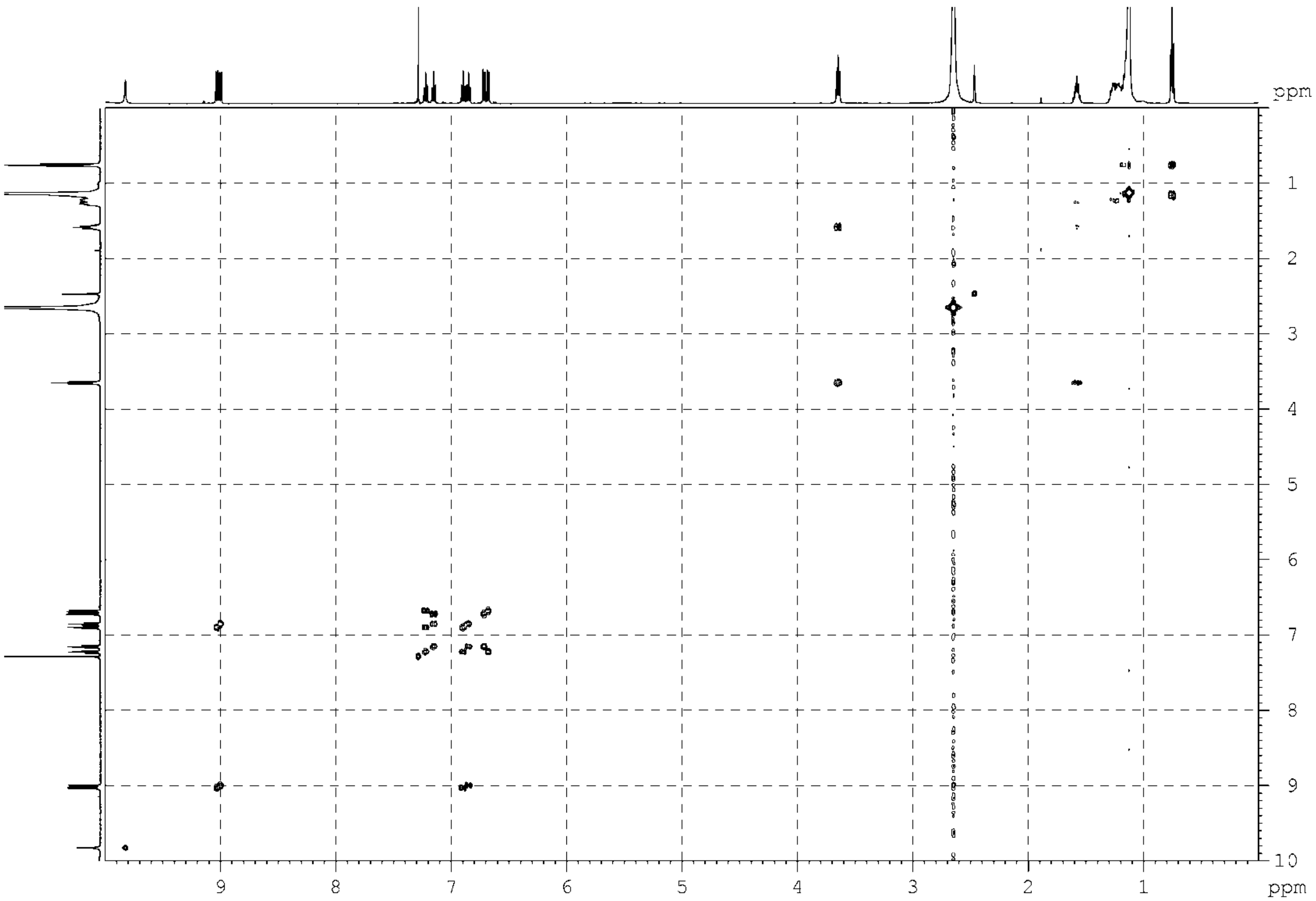

Figure S26: 2D $^{1}H,^{1}H$ COSY NMR spectra of **3**.

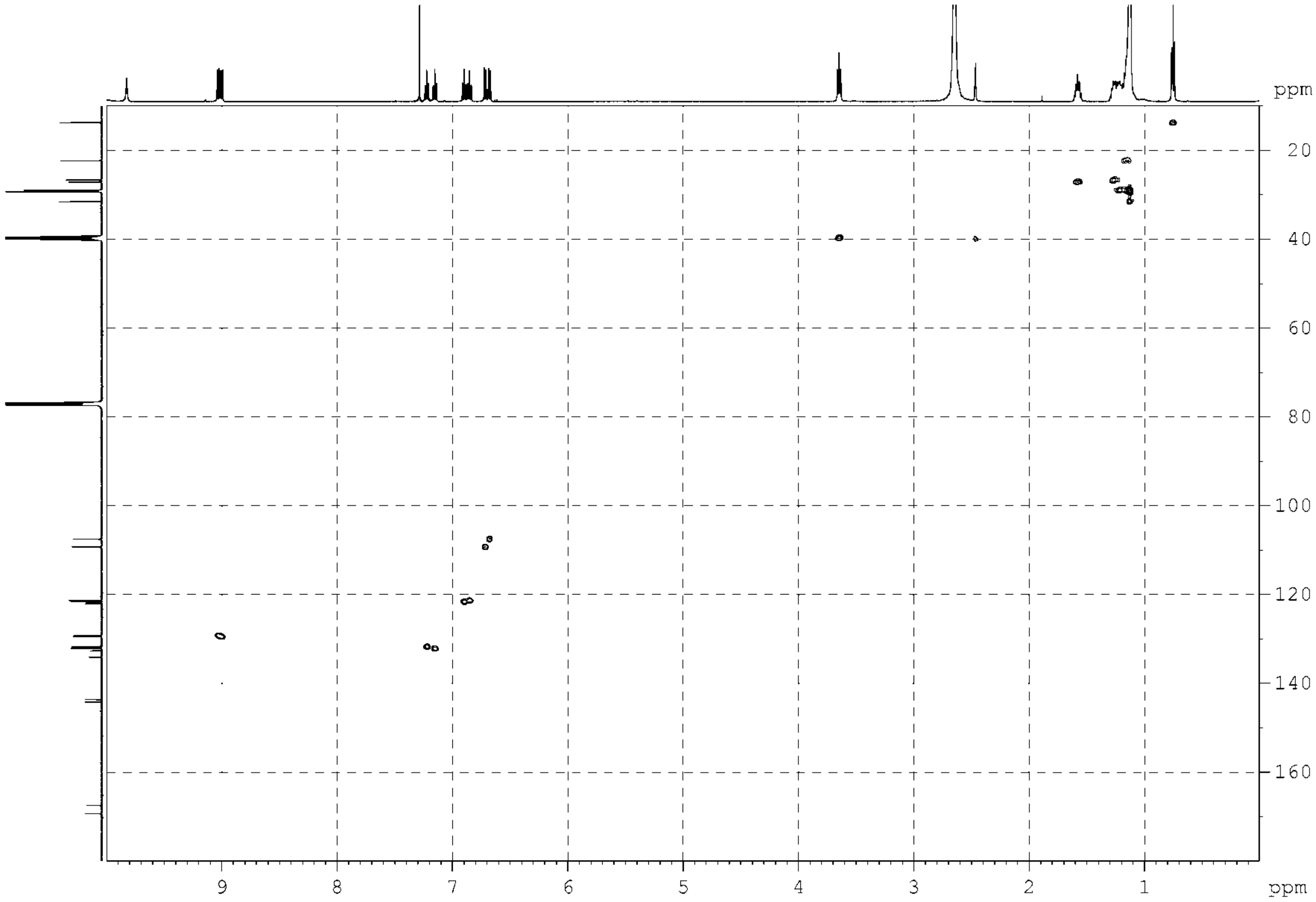

Figure S27: 2D ^{1}H,^{13}C HSQC NMR spectra of **3**.

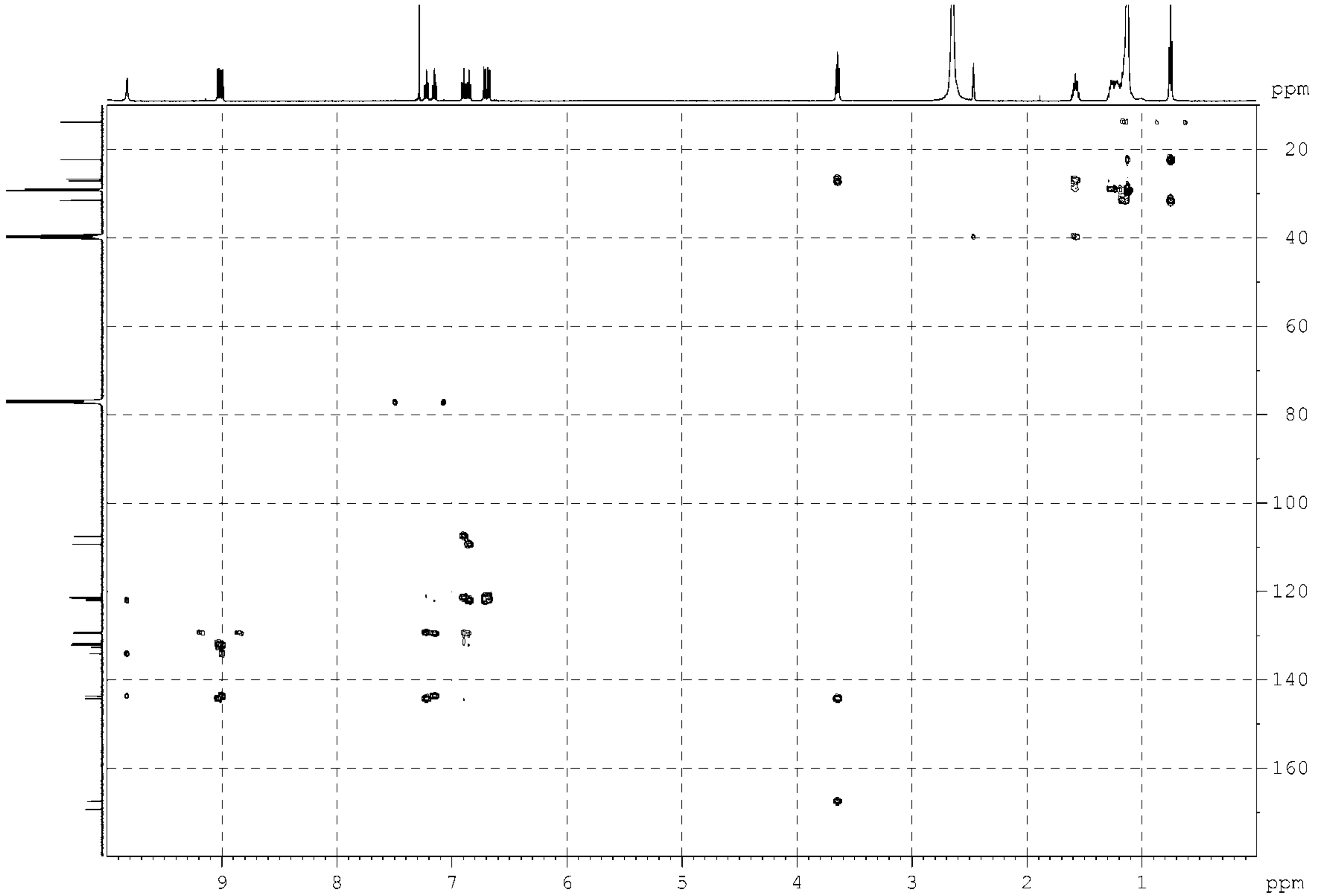

Figure S28: 2D ^{1}H,^{13}C HMBC NMR spectra of **3**.

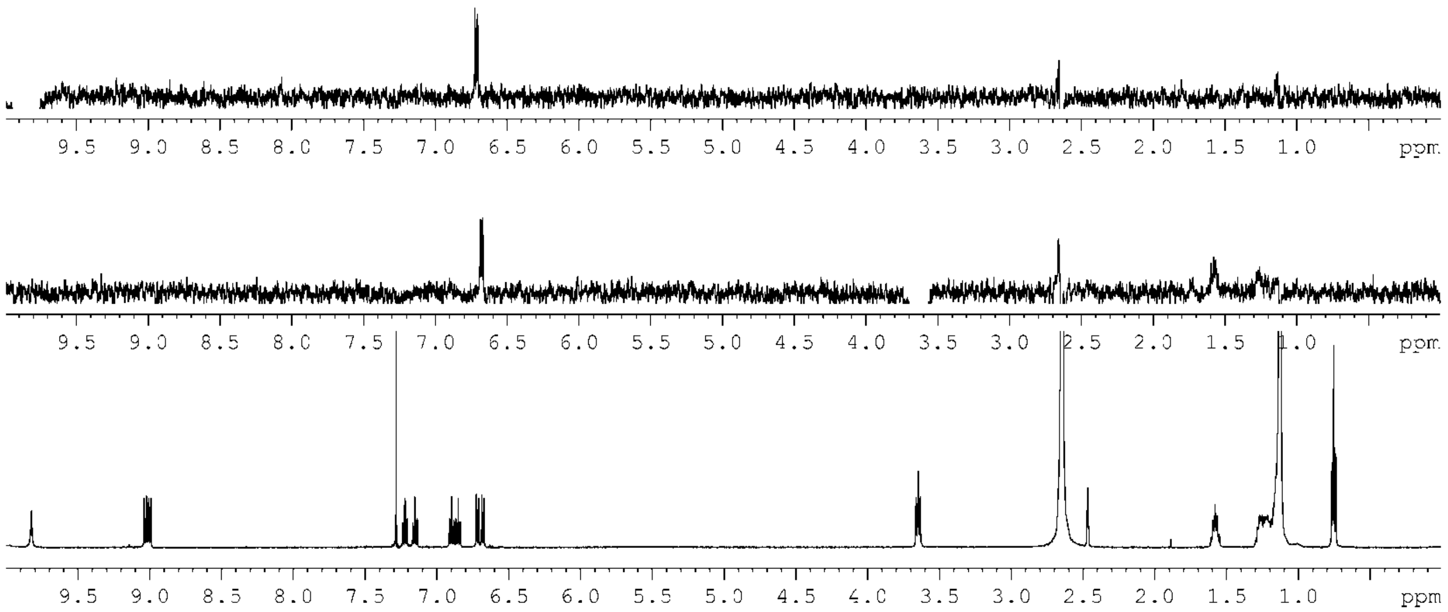

Figure S29: 1D ^{1}H and ^{1}H DPFGNOE NMR spectra of **3**.

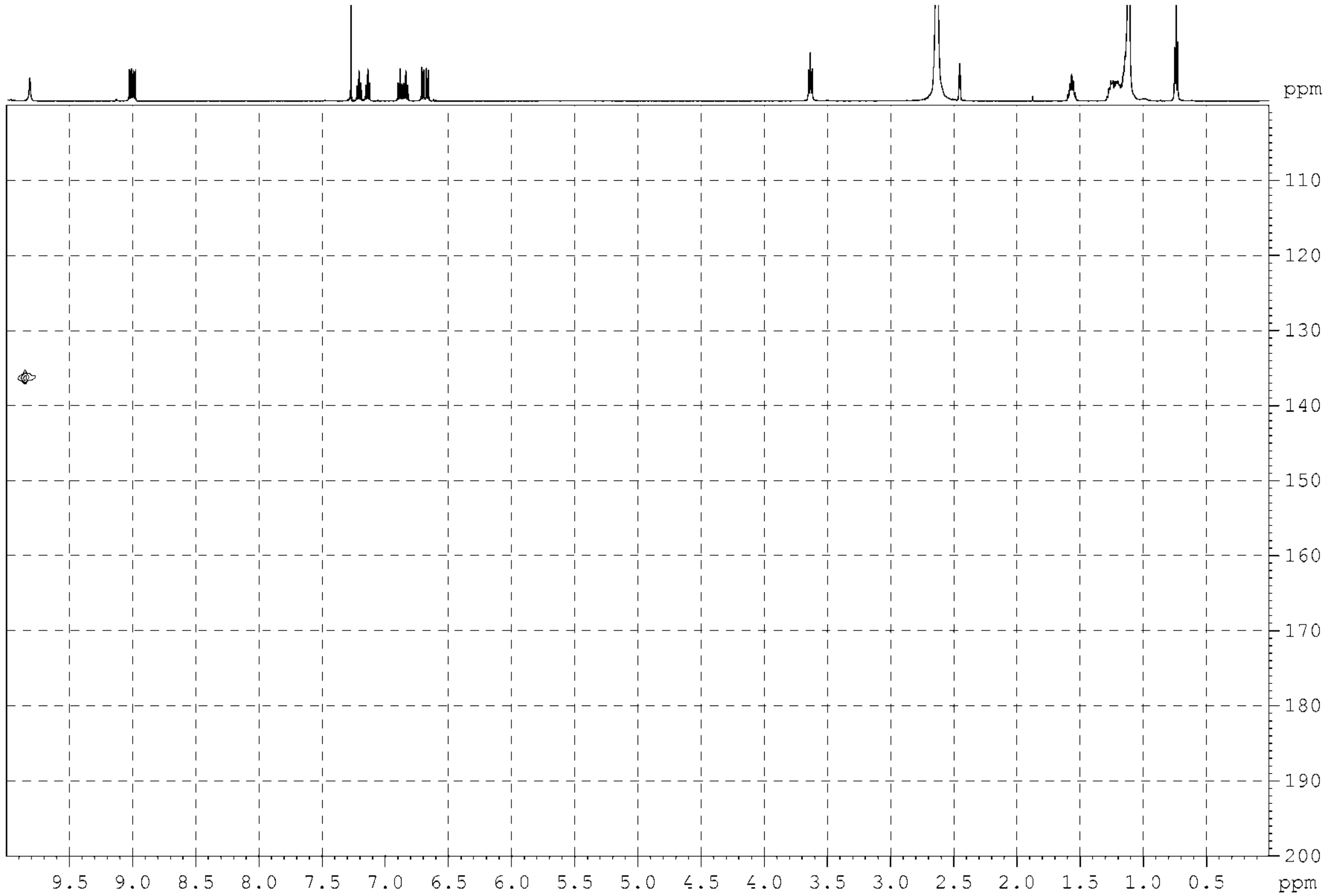

Figure S30: 2D $^{1}H,^{15}N$ HSQC NMR spectra of **3**.

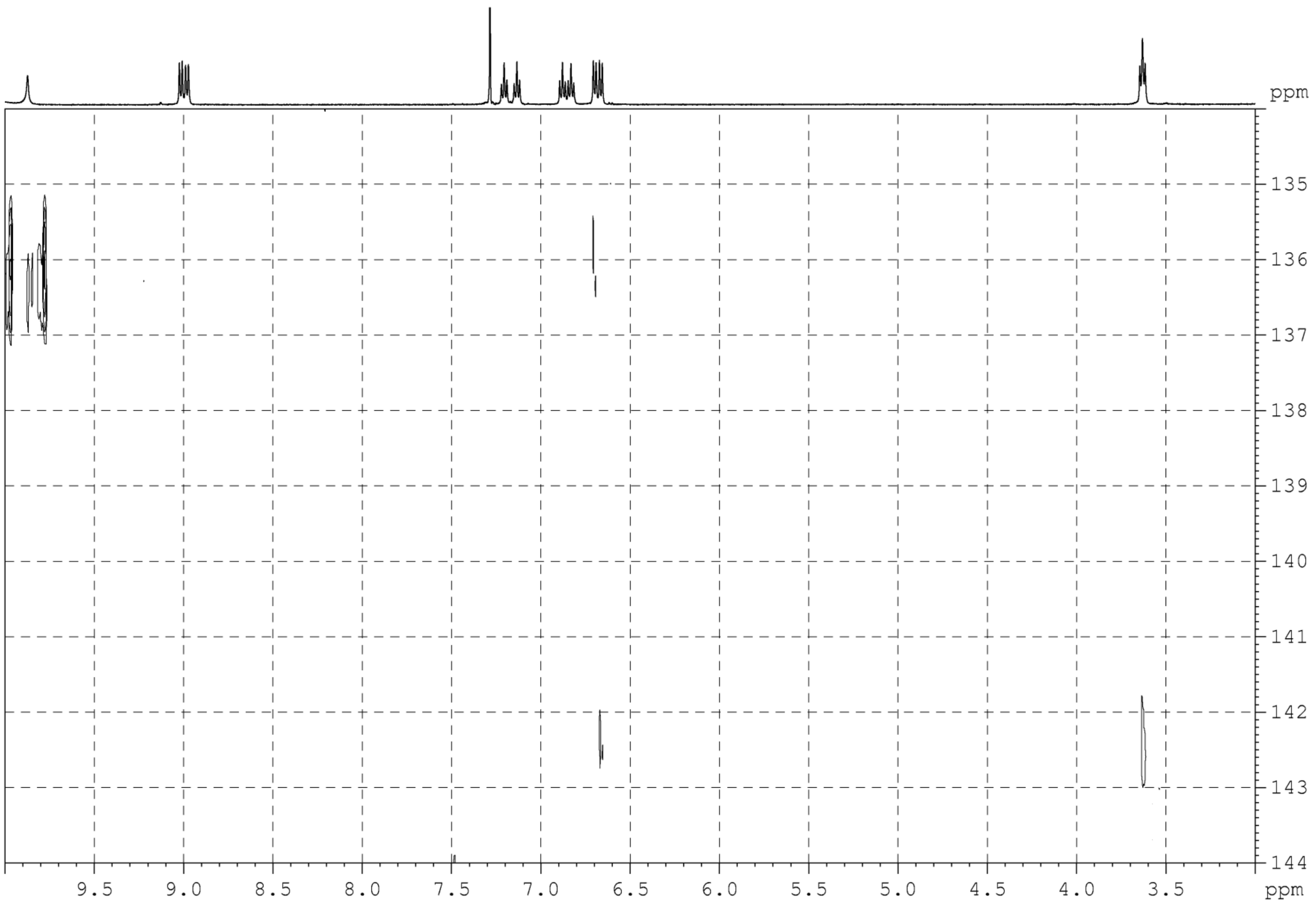

Figure S31: 2D $^{1}H,^{15}N$ HMBC NMR spectra of **3**.

Table S6: Calculated (B3LYP/6-31G(d)//B3LYP/6-31G(d)) for **3'** and experimental for **3** ^{13}C and ^{15}N (in $CDCl_3$/DMSO-d_6 (9:1)) CSs.

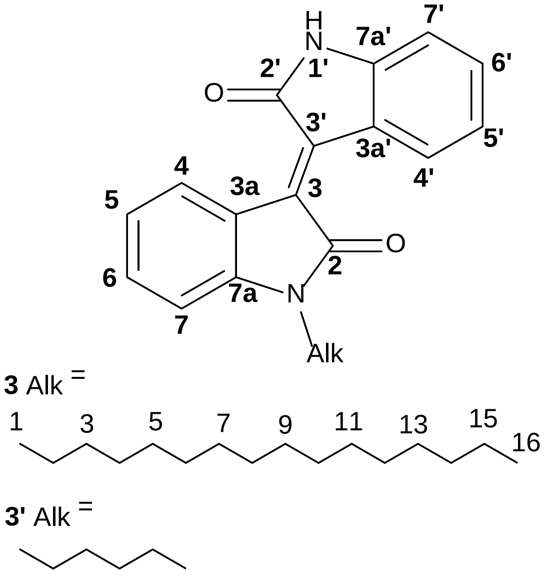

NUCL	GIAO CS, ppm	Expt, CS, ppm
C5	116.8353	121.64
C6	127.4504	131.76
C7	101.9643	107.5
C7a	139.4555	144.22
C3a	118.3801	121.32
C4	128.0972	129.3
C2	161.3018	167.5
C3	132.0753	132.62
C1-Alk	42.186	39.66
C5'	116.7246	121.3
C6'	127.14	132.12
C7'	102.571	109.3
C7a'	136.6343	143.67
C3a'	119.1201	121.99
C4'	128.1603	129.47
C2'	160.659	169.34
C3'	131.0128	134.11
C2-Alk	31.2391	27.08
C3-Alk	31.483	26.63
C14-Alk	35.5828	31.49
C15-Alk	27.6541	22.25
C16-Alk	17.5101	13.73
N1	158.3325	142.5
N1'	145.4893	136.2

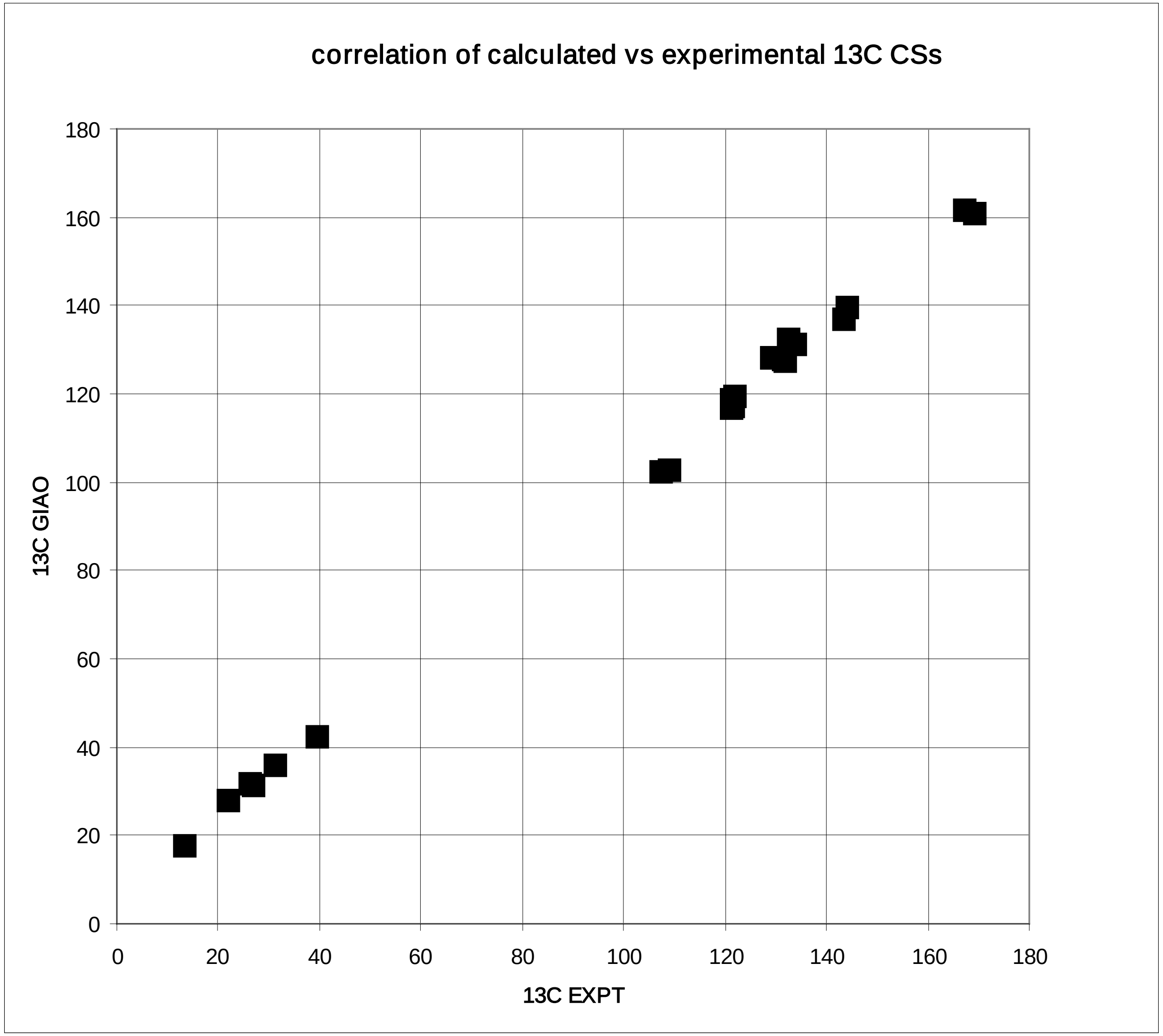

Figure S32: Correlations of calculated (B3LYP/6-31G(d)//B3LYP/6-31G(d), for **3’**) versus experimental ^{13}C chemical shifts for **3**.

References

1. Cambrige structural database system. Version 1.17 Cambrige Crystallographic Data Centre, 2014.

2. Yu.K. Voronina, D.B. Krivolapov, A.V. Bogdanov, V.F. Mironov, I.A. Litvinov, An гnusual conformation of 1,1′-Dimethyl-Isoindigo in crystals. J. Str. Chem., 53(2) (2012) 413-416.

3. W. Yue, T. He, M. Stolte, M. Gsänger, F. Würthner, Cyanated шsoindigos for n-еype and фmbipolar щrganic thin film transistors, Chem. Commun., 50 (2014) 545-547.

4. A.V. Bogdanov, T.N. Pashirova, L.I. Musin, D.B. Krivolapov, L.Ya. Zakharova, V.F. Mironov, A.I. Konovalov, Novel isoindigo derivatives bearing long-chain N-alkyl substituents: synthesis and self-assemble behavior, Chem. Phys. Lett., 594 (2014**)** 69–73.

5. J. Mata, D. Varade, P. Bahadur, Aggregation behavior of quaternary salt based cationic surfactants, Thermochimica Acta 428 (2005) 147-155.

6. J.R. McElhanon, T. Zifer, S.R. Kline, D.R. Wheeler, D.A. Loy, G.M. Jamison, T.M. Long, K. Rahimian, B.A. Simmons, Thermally cleavable surfactants based on furan-maleimide Diels – Alder adducts, Langmuir 21 (8) (2005) 3259-3266.
